# Adjunctive Oral Treatment Improves Helicobacter pylori Eradication Without Evidence of a Dose-Response Gradient: An Updated Meta-Analysis Challenging the Oral Reservoir Hypothesis

**DOI:** 10.7759/cureus.117737

**Published:** 2026-10-10

**Authors:** Mihai Stefan Benchea, Andreea-Adriana Neamtu, Ciprian-Nicusor Solomon, Emilia Albinita Cuc, Mihail Pantor, Ovidiu Pop

**Affiliations:** 1 Faculty of Medicine and Pharmacy, University of Oradea, Oradea, ROU; 2 Research Centre for Pharmaco-Toxicological Evaluation, "Victor Babeș" University of Medicine and Pharmacy Timișoara, Timișoara, ROU; 3 1st Clinic of Obstetrics and Gynecology, "Pius Brînzeu" Clinical County Emergency Hospital Timișoara, Timișoara, ROU

**Keywords:** chlorhexidine, dental plaque, eradication rate, helicobacter pylori, meta-analysis, oral hygiene, oral microbiome, periodontal therapy, recurrence, scaling and root planing

## Abstract

Standard antibiotic regimens for *Helicobacter pylori* are failing more often as resistance rises, and the oral cavity has long been proposed as an extragastric reservoir that seeds gastric recolonization. If so, treating the mouth should improve gastric eradication, a testable clinical claim rather than a correlation. We therefore pooled the randomized evidence on adjunctive periodontal and oral hygiene treatment identified through our search strategy and tested the reservoir hypothesis rather than assuming it.

We included randomized and quasi-randomized controlled trials in which adults or children receiving standard eradication therapy for confirmed gastric *H. pylori* infection were allocated to receive, or not receive, an adjunctive periodontal or oral hygiene intervention. Trials were identified by citation searching of three previous syntheses and by targeted searches for trials published after August 2015, without language restriction. The primary outcome was eradication confirmed at the first post-treatment assessment, at least four weeks after therapy; the co-primary outcome was absence of recurrence at six months or later. We pooled odds ratios and risk ratios using random-effects models with the Hartung-Knapp adjustment, assessed risk of bias with the second version of the Cochrane risk-of-bias tool, and rated certainty using the Grading of Recommendations Assessment, Development, and Evaluation (GRADE) approach. Subgroup analyses by intervention intensity, region, publication period, and source verifiability were prespecified, and small-study effects were examined by funnel plot, Egger and Begg tests, and trim-and-fill.

Eleven trials contributed data. For eradication, 10 trials, including 1,575 participants, gave a pooled odds ratio of 2.84 (95% confidence interval 1.84 to 4.39; p = 0.0004) with modest heterogeneity (I-squared 34.4%, prediction interval 1.18 to 6.87), corresponding to eradication in 87.1% versus 72.0% and a number needed to treat of six. For recurrence, four trials including 999 participants gave a pooled odds ratio for remaining free of infection of 4.64 (95% confidence interval 2.06 to 10.45); the same trials pooled as risk ratios gave 1.63 (95% confidence interval 0.86 to 3.09), and both are reported with equal prominence because the finding does not survive the change of scale. There was no evidence of small-study effects (Egger p = 0.51; none imputed by trim-and-fill). No gradient with intervention intensity was detected: an antiseptic mouthrinse alone performed at least as well as full-mouth scaling and root planing (test for subgroup differences p = 0.19), although the stratum-level confidence intervals were wide, and the largest single effect in the dataset came from a placebo-controlled trial of an antiseptic mouthrinse with no mechanical component. No trial was at low overall risk of bias.

Adjunctive oral treatment is consistently associated with better eradication, but no evidence of a dose-response gradient was detected, which does not support simple mechanical removal of an oral reservoir as the explanation. Certainty was moderate for eradication, downgraded for risk of bias, and low for recurrence, downgraded for risk of bias and imprecision.

## Introduction and background

*Helicobacter pylori *and the problem of treatment failure

*Helicobacter pylori* infects an estimated 43.9% of adults worldwide [1] and is the dominant modifiable cause of gastric adenocarcinoma, to which approximately three-quarters of the 15.6 million lifetime gastric cancers projected for the 2008 to 2017 birth cohort are attributable [2]. Eradication reduces gastric cancer incidence in infected individuals (pooled risk ratio 0.64, 95% confidence interval 0.48 to 0.84, eight randomized trials) [3], so failure of first-line therapy is a missed opportunity for cancer prevention.

That failure is becoming more common. Global clarithromycin resistance has risen from 29.1% in 2015 to 2019 to 36.5% in 2020 to 2023 [4], and guidance has moved from clarithromycin triple therapy to bismuth quadruple, concomitant and susceptibility-guided regimens [5,6]. Almost all of this effort has targeted the antibiotic regimen itself.

The oral reservoir hypothesis

*Helicobacter pylori* DNA is detectable in dental plaque, saliva and periodontal pockets, most often in plaque and with deeper probing depths [7]. Because eradication regimens reach the gastric mucosa far more reliably than the oral biofilm, the extragastric reservoir hypothesis proposes that oral organisms survive systemic therapy and reseed the stomach [8,9]. The observational literature on this association is saturated, with at least nine meta-analyses since 2019 whose conclusions conflict: pooled odds ratios range from 2.47 [10] to 4.28 [11], the review with the strictest inclusion criteria found no significant association [12], and in another the association disappeared within diagnostic-method subgroups [13]. Such studies cannot show that the mouth is the source of gastric infection.

The interventional evidence

If the mouth is a reservoir, treating it should improve outcomes in the stomach, which is a testable clinical claim rather than a correlation. An early meta-analysis pooled three trials for recurrence only [14]. A Cochrane review pooled seven trials (691 participants), with odds ratios of 2.15 (95% confidence interval 1.47 to 3.14) for eradication and 3.60 (95% confidence interval 2.11 to 6.15) for absence of recurrence [15], but its search closed in August 2015, and it has not been updated. A non-Cochrane update covered trials to 2020 [16], and the most recent synthesis (2025) was restricted to English-language reports from 2015 onward and performed no quantitative synthesis [17]. At least three eligible randomized trials have been published since the Cochrane search [18-20].

Rationale and objectives

Two features of this literature have not been examined. First, the pooled interventions range from a twice-daily antiseptic mouthrinse to single-stage full-mouth disinfection. If the mechanism is mechanical disruption of an oral biofilm reservoir, the effect should increase with the thoroughness of that disruption, so a dose-response gradient directly tests the proposed mechanism. Second, the reported effects are large for a co-intervention of modest biological reach, and the trials are concentrated in one country and partly published in journals not indexed internationally, a configuration in which small-study effects are common and rarely tested for.

The answer matters for practice and research. If the benefit is real and mechanical, periodontal therapy could be offered as a routine adjunct to eradication; if it is real but not mechanical, a simpler and more scalable intervention may suffice, and trials should be designed to identify the active component; if it is an artifact of small or biased trials, resources are better directed elsewhere. Our objectives were accordingly threefold: to update the pooled estimates for eradication and for recurrence using the randomized evidence identified through the present search strategy, without language restriction; to determine whether the effect varies with the intensity of the oral intervention, and thereby to test the mechanism rather than assume it; and to interrogate the pooled estimate for small-study effects and for dependence on trials whose primary reports are difficult to obtain.

## Review

Methods

Protocol, Registration, and Reporting

This review was conducted in accordance with the Preferred Reporting Items for Systematic Reviews and Meta-Analyses (PRISMA) 2020 statement [21], and the completed 27-item checklist is provided in Appendix 1. Eligibility criteria, outcomes, and all subgroup and sensitivity analyses were specified in a written protocol before any data were pooled, and were not altered subsequently; the complete analysis code and the extracted dataset are provided in Appendices 2, 6, 7. Because the review used only aggregate data from published studies, Institutional Review Board approval was not required.

Eligibility Criteria

This analysis included randomized and quasi-randomized controlled trials enrolling participants of any age with gastric *H. pylori *infection confirmed by a validated method, in which the intervention group received a defined periodontal or oral hygiene intervention in addition to a standard eradication regimen, and the control group received the same regimen alone or with no oral co-intervention. Eligible oral interventions included supragingival scaling, subgingival scaling, scaling and root planing, full-mouth disinfection, professional plaque control, structured oral hygiene instruction, antiseptic mouth rinse, and tongue cleaning.

This analysis excluded observational and cross-sectional association studies, trials in which the adjunct was an additional systemic antibiotic rather than an oral intervention, trials of oral *H. pylori *clearance that included no systemic eradication regimen and no gastric outcome, studies of periodontal surgery, and duplicate reports of a single cohort.

Outcomes

The primary outcome was eradication of gastric *H. pylori* confirmed at the first scheduled post-treatment assessment, at least four weeks after completion of therapy, by urea breath test, stool antigen test, histology, rapid urease test, culture, or polymerase chain reaction. Assessment was at four to eight weeks in every trial except one, in which it was at three months [22]. The co-primary outcome was absence of recurrence at six months or later following documented eradication. Prespecified secondary outcomes were clearance of oral *H. pylori,* periodontal clinical parameters, dyspepsia symptom scores, adverse events, and treatment adherence.

Information Sources and Search

Eligible trials were identified by citation searching rather than by a de novo multi-database search. We examined the reference lists and the included- and excluded-study tables of the three previous syntheses of this question [15-17], which, between them, had searched Medical Literature Analysis and Retrieval System Online (MEDLINE), Embase, the Cochrane Central Register of Controlled Trials, Scopus, Web of Science, and the Chinese-language databases (China National Knowledge Infrastructure and Wanfang), and we then searched PubMed and publisher websites for eligible randomized trials published after the Cochrane search closed in August 2015. Searching was completed in July 2026. No language restriction was applied at any stage, and trials published in Chinese-language journals were retained.

This identification strategy is the principal methodological limitation of the review. Its practical consequence is that any eligible trial missed by all three previous syntheses and not retrievable through PubMed would also be missed here. Its practical strength is that the three previous syntheses collectively covered the major international and Chinese-language databases, so the pooled evidence base is larger than that of any of them individually.

Study Selection and Data Extraction

Records were deduplicated and screened independently by two reviewers, first by title and abstract and then in full text, with disagreements resolved by a third. Two reviewers independently extracted, into a piloted form, the study identifier, country, design, method of sequence generation and allocation concealment, blinding, number randomized and analyzed in each arm, participant age and sex, baseline periodontal status and how it was defined, the full composition and timing of the oral intervention, the eradication regimen and its duration, the outcome definition and the method by which it was confirmed, the timing of outcome assessment, losses to follow-up, and funding and conflicts of interest.

Where a trial reported percentages without integer counts, counts were derived, and every derivation is declared alongside the study characteristics. For seven trials, arm-level event data were taken from the published forest plots and characteristics tables of the Cochrane review rather than from the primary reports [15]; five of these are Chinese-language reports in journals that are not indexed internationally and could not be obtained. The primary report of one of the other two [22] could not be obtained, and that of the other could not be reconciled with the transcribed counts, which are reported in the Limitations and examined in a post hoc sensitivity analysis. This dependence is reported as a limitation and was examined in a prespecified sensitivity analysis. As a check on extraction fidelity, the subset of trials taken from that source was pooled in isolation and reproduced the published summary counts and pooled estimate exactly (Appendix 3).

Risk of Bias and Certainty of Evidence

Two reviewers independently assessed each trial with the second version of the Cochrane risk-of-bias tool for randomized trials, covering the randomization process, deviations from intended interventions, missing outcome data, measurement of the outcome, and selection of the reported result, with an overall judgment of low risk, some concerns, or high risk [23]. Domain-level judgments and their supporting statements are given in Appendix 4. Certainty of evidence for each outcome was rated using the Grading of Recommendations Assessment, Development, and Evaluation (GRADE) approach [24] and is reported with the Results.

Statistical Analysis

Dichotomous outcomes were summarized as odds ratios and, for interpretability, also as risk ratios, each with 95% confidence intervals. Estimates were pooled using the DerSimonian-Laird random-effects model with the Hartung-Knapp-Sidik-Jonkman adjustment to the standard error, which yields better coverage than the conventional normal approximation when the number of trials is small; the unadjusted random-effects and fixed-effect estimates are reported alongside for comparison. Heterogeneity was quantified by the Cochran Q statistic, the I-squared statistic, and the between-study variance tau-squared, and a 95% prediction interval was computed for each pooled estimate. A continuity correction of 0.5 was applied only where an arm contained no events.

One trial contributed two intervention arms sharing a single control group [25]; the arms were combined for the primary analysis and, for the intensity-stratified analysis, were entered separately with the shared control group divided between them. Two trials also enrolled a non-randomized group of participants who tested negative for oral *H. pylori *and received eradication therapy alone [19,25]; because allocation to that group was by oral infection status rather than at random, it was excluded, and only the randomized groups were analyzed. A second four-arm trial allocated participants to an antiseptic mouth rinse or to a saline mouth rinse within two strata defined by the presence or absence of periodontal disease, with periodontal treatment given to every participant in the periodontal-disease stratum irrespective of allocation [20]; only the randomized mouth rinse contrast was used, pooled across the two strata, and the trial’s non-randomized comparison between the strata was not entered into any analysis.

Prespecified subgroup analyses examined intervention intensity, categorized as antiseptic mouth rinse or oral hygiene instruction alone, professional supragingival and subgingival scaling, or scaling and root planing with full-mouth disinfection; geographical region; and period of publication relative to the Cochrane search. Prespecified sensitivity analyses excluded trials at high overall risk of bias, restricted the analysis to trials whose arm-level counts came from a directly obtained primary report, and removed each trial in turn. Small-study effects were examined by a funnel plot with a 95% pseudo-confidence region, the Egger regression test, the Begg rank correlation test, and the Duval and Tweedie trim-and-fill procedure. Analyses were performed in Python 3.11 (Python Software Foundation, Fredericksburg, VA) using NumPy, SciPy, and Matplotlib; the analysis code and the extracted dataset are provided in Appendices 2, 6, 7.

Results

Study Selection and Characteristics

The study selection process is summarized in Figure 1. Eleven randomized or quasi-randomized trials met the inclusion criteria. Ten trials contributed to the eradication analysis and four to the recurrence analysis, with three trials contributing to both. Characteristics of the included trials are given in Table 1. One trial [18] contributed 700 participants, 44% of the eradication analysis and 70% of the recurrence analysis; the remaining trials analyzed 43 to 168 participants each.

**Figure 1:**
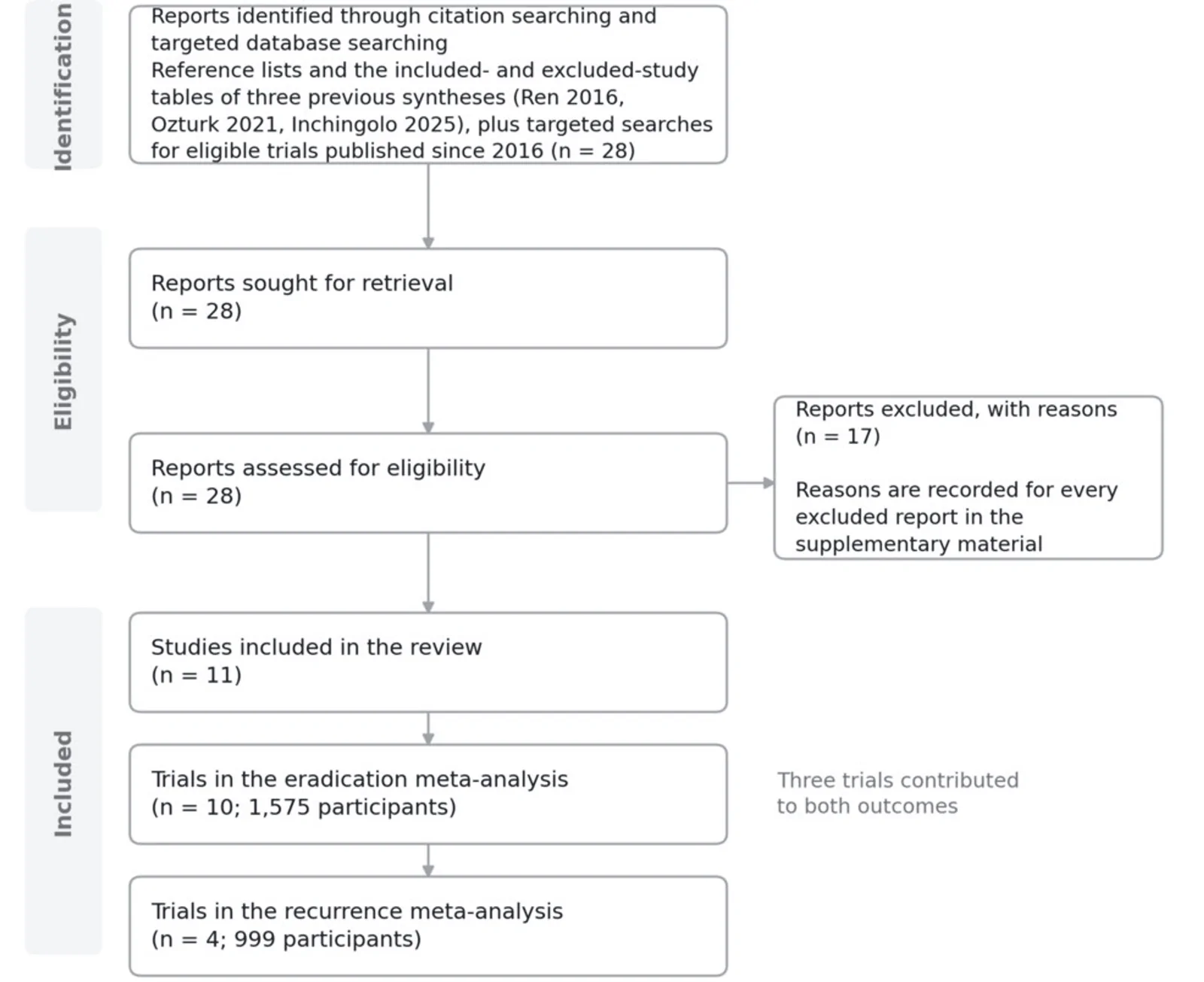
PRISMA 2020 flow diagram for study identification, screening and inclusion. Reports were identified from the reference lists and the included- and excluded-study tables of three previous syntheses of this question: Ren 2016 [15], Ozturk 2021 [16], and Inchingolo 2025 [17], together with targeted searches for eligible randomized trials published after the Cochrane search closed in August 2015. Reasons for exclusion are recorded for every excluded report in Appendix 5. PRISMA: Preferred Reporting Items for Systematic Reviews and Meta-Analyses

**Table 1:** Characteristics of the 11 included trials. Trials are listed in order of publication. The intensity stratum in parentheses is the category used in the intensity-stratified analysis. Participants analyzed are the total of both randomized arms; for Song 2013 [25], the two intervention arms are counted together, and for Song 2013 [25], Urrutia-Baca 2024 [20] and Behfarnia 2025 [19], groups that were not formed by randomization are excluded, as described in the Methods. Outcome assessment was at four to eight weeks after therapy in every trial except Zaric 2009 [22], in which it was at three months. Arm-level counts and the provenance of each are given in Appendix 2. PPI: proton pump inhibitor

Trial	Country	Design	Control group	Oral intervention (intensity stratum)	Eradication regimen	Outcomes contributed (participants analyzed)
Yuan 2003 [26]	China	Randomized	Eradication therapy alone	Initial periodontal therapy with supragingival and subgingival scaling (professional scaling)	PPI-based triple therapy	Eradication (64); recurrence (64)
Lv 2006 [27]	China	Randomized	Eradication therapy alone	Supragingival and subgingival scaling with Rhizoma Coptidis aerosol (professional scaling)	PPI-based triple therapy	Eradication (65)
Jin 2007 [28]	China	Randomized	Eradication therapy alone	Initial periodontal therapy with supragingival and subgingival scaling (professional scaling)	PPI-based triple therapy	Eradication (87); recurrence (87)
Zaric 2009 [22]	Serbia	Randomized	Eradication therapy alone	Scaling and root planing (scaling and root planing or full-mouth disinfection)	PPI-based triple therapy	Eradication (43)
Jia 2009 [29]	China	Randomized	Eradication therapy alone	Professional plaque control (not stratified; recurrence only)	PPI-based triple therapy	Recurrence (148)
Liu 2012 [30]	China	Randomized	Eradication therapy alone	Supragingival and subgingival scaling (professional scaling)	PPI-based triple therapy	Eradication (152)
Song 2013 [25]	China	Randomized, three arms	Eradication therapy alone (shared by both intervention arms)	Arm 1: mouth rinse (mouth rinse or oral hygiene alone); arm 2: mouth rinse with periodontal treatment (professional scaling)	PPI-based triple therapy	Eradication (168)
Wang 2014 [31]	China	Randomized	Eradication therapy alone	Antiseptic mouthwash (mouth rinse or oral hygiene alone)	PPI-based triple therapy	Eradication (132)
Tongtawee 2019 [18]	Thailand	Randomized	Eradication therapy alone	Scaling and root planing or full-mouth disinfection with chlorhexidine (scaling and root planing or full-mouth disinfection)	PPI-based triple therapy	Eradication (700); recurrence (700)
Urrutia-Baca 2024 [20]	Mexico	Quasi-randomized (alternation by order of consent), stratified by periodontal status	Matched saline placebo rinse	Neutral electrolyzed water mouth rinse (mouth rinse or oral hygiene alone)	Non-bismuth quadruple (concomitant) therapy	Eradication (100)
Behfarnia 2025 [19]	Iran	Randomized	Eradication therapy alone	Nonsurgical periodontal therapy, scaling and root planing (scaling and root planing or full-mouth disinfection)	Bismuth quadruple therapy	Eradication (64)

The included trials were conducted in China (seven trials), Serbia, Thailand, Mexico, and Iran and were published between 2003 and 2025. Arm sizes ranged from 21 to 350 participants, and the trials together contributed 1,723 analyzed participants; 1,575 contributed to the eradication analysis and 999 to the recurrence analysis. Eradication regimens were predominantly one-week or two-week proton pump inhibitor-based triple therapy, with non-bismuth quadruple concomitant therapy in one trial [20] and bismuth quadruple therapy in the most recent trial [19]. Outcome confirmation used carbon-13 or carbon-14 urea breath testing in six trials, stool antigen testing in two, rapid urease testing or histology in one, and polymerase chain reaction in two.

The oral interventions differed substantially. Six delivered professional supragingival and subgingival scaling, generally combined with oral hygiene instruction and a mouthwash [25-30]. Three delivered an antiseptic mouth rinse or structured oral hygiene alone [20,25,31]. Three delivered scaling and root planing or single-stage full-mouth disinfection with chlorhexidine irrigation of periodontal pockets [18,19,22]. One of these trials contributed to the recurrence outcome only and therefore does not enter the intensity-stratified analysis [29], and one contributed two intervention arms of different intensity and is counted in two categories [25]. Only one trial used an inert placebo rinse in the control arm [20]. In the remainder, the control group received eradication therapy alone with no matched oral procedure. Timing also varied, from interventions concurrent with and ending with the antibiotic course to plaque control continued for six months after eradication.

Risk of Bias

Risk-of-bias judgments are shown in Figure 2. No trial was judged at low overall risk of bias. Eight trials raised some concerns, most often because the method of sequence generation and allocation concealment was not described and because no prespecified protocol was available against which to check the reported analysis. Three trials were at high risk: one because a substantial and unquantified number of participants did not complete the study, and neither the number nor the reasons were reported [22]; one because all participants were reported as free of gastric infection after eradication therapy, an implausible 100% success rate that raises concern about outcome measurement [29]; and one because allocation followed the order in which participants consented, with odd numbers assigned to the control rinse and even numbers to the active rinse, a deterministic and unconcealable sequence [20]. Blinding of participants to an oral intervention is intrinsically difficult, but the outcomes are laboratory-confirmed and objective, so measurement of the outcome was judged at low risk in most trials.

**Figure 2:**
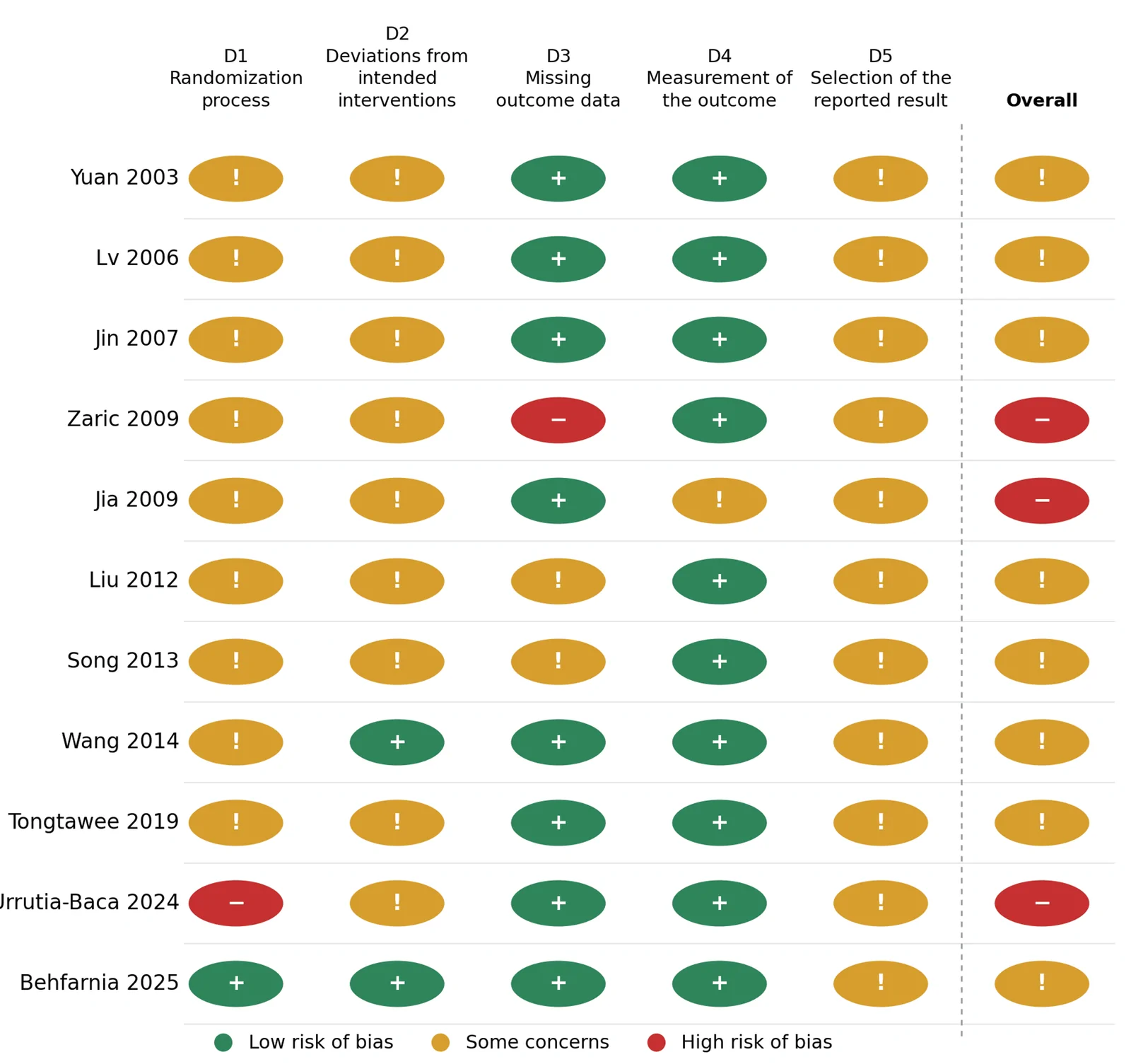
Risk of bias in the 11 included randomized trials, assessed with the second version of the Cochrane risk-of-bias tool. Green denotes low risk, amber denotes some concerns, and red denotes high risk. The trials shown, in the order plotted, are Yuan 2003 [26], Lv 2006 [27], Jin 2007 [28], Zaric 2009 [22], Jia 2009 [29], Liu 2012 [30], Song 2013 [25], Wang 2014 [31], Tongtawee 2019 [18], Urrutia-Baca 2024 [20], and Behfarnia 2025 [19]. D1: randomization process; D2: deviations from intended interventions; D3: missing outcome data; D4: measurement of the outcome; D5: selection of the reported result. The tool itself is described in [23]; domain-level judgments with their supporting statements are given in Appendix 4.

Eradication of Gastric Helicobacter pylori

Ten trials, including 1,575 participants, reported eradication (Figure 3). Eradication was achieved in 716 of 822 participants (87.1%) allocated to adjunctive oral treatment and in 542 of 753 (72.0%) allocated to eradication therapy alone. The pooled odds ratio was 2.84 (95% confidence interval 1.84 to 4.39; p = 0.0004). Heterogeneity was modest and not statistically significant (Q = 13.73, df = 9, p = 0.13; I-squared 34.4%; tau-squared 0.112), and the 95% prediction interval, from 1.18 to 6.87, excluded the null, indicating that a future trial in a comparable setting would be expected to show benefit. Expressed as a risk ratio, the pooled estimate was 1.27 (95% confidence interval 1.08 to 1.49), although on that scale heterogeneity was substantial (I-squared 68.0%), and the prediction interval crossed the null. Applying the pooled odds ratio to the observed control-group risk of 72.0% corresponds to an absolute increase of 16.0 percentage points and a number needed to treat of six. Because the odds ratio scale compresses at high baseline risk, the absolute benefit is markedly smaller where eradication rates are already high: at a baseline of 90%, the number needed to treat rises to 16.

**Figure 3:**
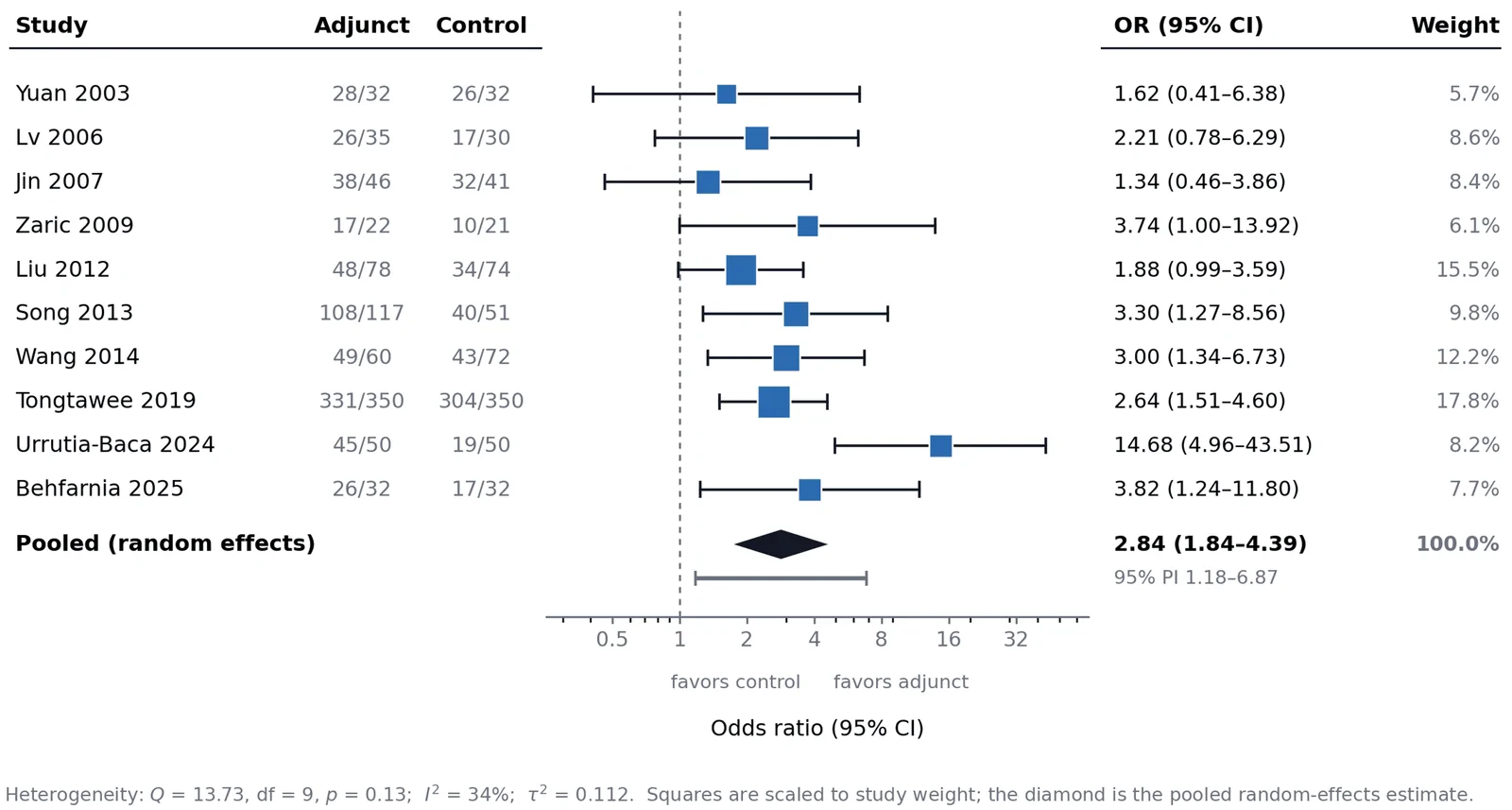
Forest plot for eradication of gastric Helicobacter pylori. Squares are scaled to study weight, and the diamond is the pooled random-effects estimate; the horizontal bar below the diamond is the 95% prediction interval. The trials shown, in the order plotted, are Yuan 2003 [26], Lv 2006 [27], Jin 2007 [28], Zaric 2009 [22], Liu 2012 [30], Song 2013 [25], Wang 2014 [31], Tongtawee 2019 [18], Urrutia-Baca 2024 [20], and Behfarnia 2025 [19]. The two intervention arms of Song 2013 [25] are combined into a single trial-level estimate.

Every individual trial estimate favored the adjunct, with point estimates between 1.34 and 14.68; six of the 10 reached conventional significance individually. Omitting any single trial changed the pooled odds ratio only between 2.45 and 3.07, and the interval excluded the null in every case. Almost all of the observed heterogeneity was contributed by one trial: omitting Urrutia-Baca 2024 returned the pooled estimate to 2.45 (95% confidence interval 1.74 to 3.44) with an I-squared of 0%, whereas omitting any other trial left I-squared between 32% and 42%.

Absence of Recurrence

Four trials, including 999 participants, reported recurrence at six to 12 months (Figure 4). Participants allocated to adjunctive oral treatment were more likely to remain free of infection, with a pooled odds ratio of 4.64 (95% confidence interval 2.06 to 10.45; p = 0.009), modest heterogeneity (I-squared 19.7%; tau-squared 0.052), and a wide prediction interval from 1.07 to 20.16. Crude figures were 446 of 537 (83.1%) versus 330 of 462 (71.4%).

**Figure 4:**
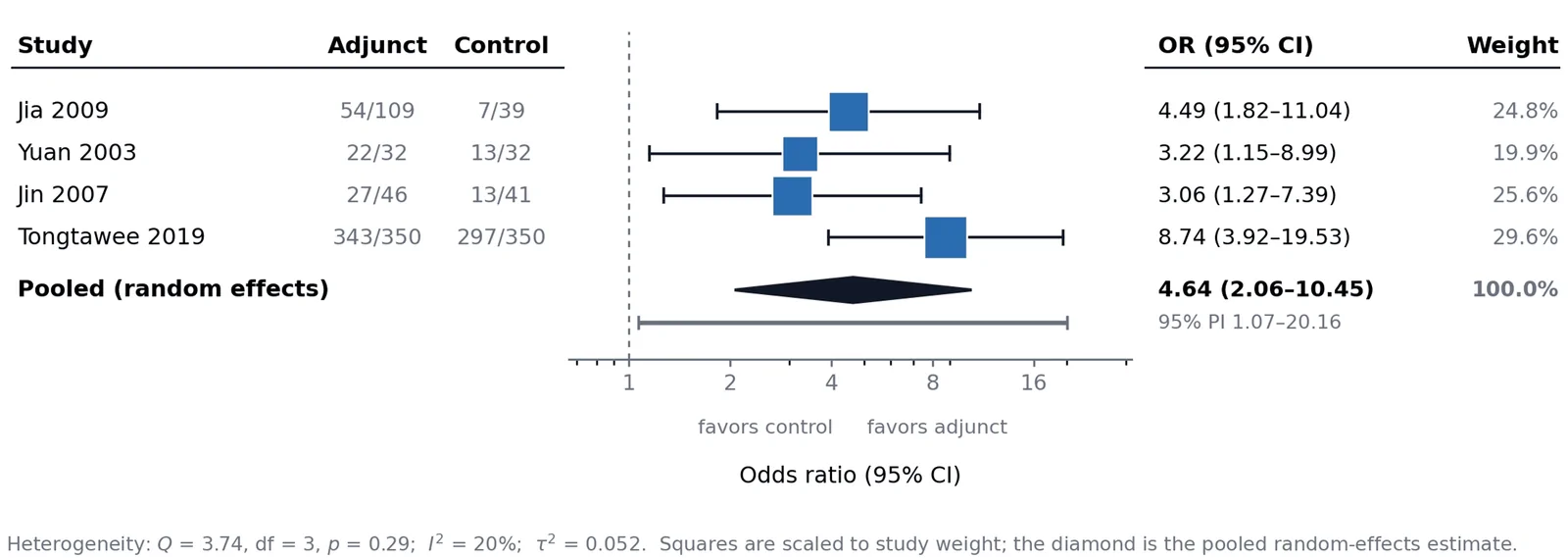
Forest plot for absence of recurrence of gastric Helicobacter pylori after successful eradication. Squares are scaled to study weight, and the diamond is the pooled random-effects estimate; the horizontal bar below the diamond is the 95% prediction interval. The trials shown, in the order plotted, are Jia 2009 [29], Yuan 2003 [26], Jin 2007 [28], and Tongtawee 2019 [18].

This estimate was not robust to the choice of effect measure. Expressed as a risk ratio and with the Hartung-Knapp adjustment, the same four trials yielded 1.63 (95% confidence interval 0.86 to 3.09; p = 0.09) with substantial heterogeneity (I-squared 73.9%). The discrepancy arises because one large trial with a very high baseline non-recurrence rate dominates the risk-ratio analysis while contributing a large odds ratio [18]. We therefore report both measures with equal prominence rather than nominating one as primary: the odds ratio was prespecified and is the measure used by the previous syntheses of this question, but an effect that changes its statistical status with the choice of scale cannot be described as established, and this instability was treated as imprecision in the certainty assessment.

One further trial reported a comparison of recurrence between its randomized groups but assessed it 16 weeks after the end of therapy, which falls short of the prespecified minimum follow-up of six months, so it contributed to the eradication analysis only [20].

Subgroup and Sensitivity Analyses

Results of all prespecified subgroup and sensitivity analyses are given in Table 2, and the tests for small-study effects are presented separately in Table 3. All 13 estimates in Table 2 favored the adjunct (odds ratios 1.93 to 4.94), and the estimate was essentially unchanged by the pooling model (2.84 with and without the Hartung-Knapp adjustment; 2.76 with a fixed-effect model). Only the two subgroups limited to three comparisons that include the placebo-controlled rinse trial [20] had confidence intervals crossing the null, and heterogeneity was confined to subgroups containing that trial (I-squared 61% to 74%); every subgroup without it had an I-squared of 0%.

**Table 2:** Subgroup and sensitivity analyses for the eradication outcome. The Reference column gives the reference numbers of the trials contributing to each analysis. The intensity strata contain 11 comparisons from 10 trials because Song 2013 [25] contributed two intervention arms of different intensity, which are entered separately, with the shared control group divided between them.

Analysis	Trials (comparisons)	Reference	Odds ratio (95% confidence interval)	I-squared
All trials, random effects with Hartung-Knapp adjustment	10	[18-20,22,25-28,30,31]	2.84 (1.84 to 4.39)	34%
All trials, random effects without adjustment	10	[18-20,22,25-28,30,31]	2.84 (1.98 to 4.09)	34%
All trials, fixed effect	10	[18-20,22,25-28,30,31]	2.76 (2.08 to 3.64)	not applicable
Intervention intensity: antiseptic mouth rinse or oral hygiene alone	3	[20,25,31]	4.87 (0.47 to 49.95)	68%
Intervention intensity: professional supragingival and subgingival scaling	5	[25-28,30]	1.93 (1.04 to 3.61)	0%
Intervention intensity: scaling and root planing or full-mouth disinfection	3	[18,19,22]	2.94 (1.05 to 8.18)	0%
Test for subgroup differences by intensity	10 (11 comparisons)	[18-20,22,25-28,30,31]	Q = 3.28, df = 2, p = 0.19	
Region: China	6	[25-28,30,31]	2.19 (1.35 to 3.54)	0%
Region: outside China	4	[18-20,22]	4.57 (1.29 to 16.23)	61%
Published before the Cochrane search closed	7	[22,25-28,30,31]	2.27 (1.46 to 3.54)	0%
Published after the Cochrane search closed	3	[18-20]	4.94 (0.52 to 46.86)	74%
Arm-level counts from a directly obtained primary report	4	[18-20,25]	4.35 (1.31 to 14.39)	61%
Excluding the two contributing trials at high overall risk of bias	8	[18,19,25-28,30,31]	2.40 (1.68 to 3.43)	0%
Leave-one-out, range across all 10 omissions	9 each	[18-20,22,25-28,30,31]	2.45 to 3.07	0 to 42%

**Table 3:** Tests for small-study effects for the eradication outcome. All three tests were applied to the 10 trials contributing to the eradication outcome, with the two intervention arms of Song 2013 [25] combined into a single trial-level estimate, and correspond to the funnel plot. Trim-and-fill was applied in the direction that publication bias would produce, in which small trials showing no benefit are presumed missing from the left of the funnel. These tests have low power with 10 trials, and their negative result constrains but does not exclude concern.

Test	Trials	Reference	Result
Egger regression intercept	10	[18-20,22,25-28,30,31]	0.95 (standard error 1.39); t = 0.68, df = 8; p = 0.51
Begg rank correlation	10	[18-20,22,25-28,30,31]	Kendall tau 0.16; p = 0.60
Duval and Tweedie trim-and-fill	10	[18-20,22,25-28,30,31]	0 studies imputed; pooled estimate unchanged at 2.84 (1.84 to 4.39)

The intensity-stratified analysis is the central test of mechanism in this review (Figure 5). Antiseptic mouth rinse or oral hygiene instruction alone yielded an odds ratio of 4.87 (95% confidence interval 0.47 to 49.95) across three comparisons; professional supragingival and subgingival scaling yielded 1.93 (95% confidence interval 1.04 to 3.61) across five; and scaling and root planing or full-mouth disinfection yielded 2.94 (95% confidence interval 1.05 to 8.18) across three. The test for subgroup differences was not significant (Q = 3.28, df = 2, p = 0.19). The ordering of the point estimates is, if anything, the reverse of what the mechanical hypothesis predicts: the least intensive category was numerically the highest and the intermediate category the lowest, although the interval around the mouth rinse estimate is very wide and no firm ranking can be drawn from three comparisons. No evidence of a dose-response gradient between the thoroughness of mechanical plaque disruption and the gastric outcome was detected. Because each stratum contains only three to five comparisons and the confidence intervals are wide, this analysis has limited power, and a modest gradient cannot be excluded.

**Figure 5:**
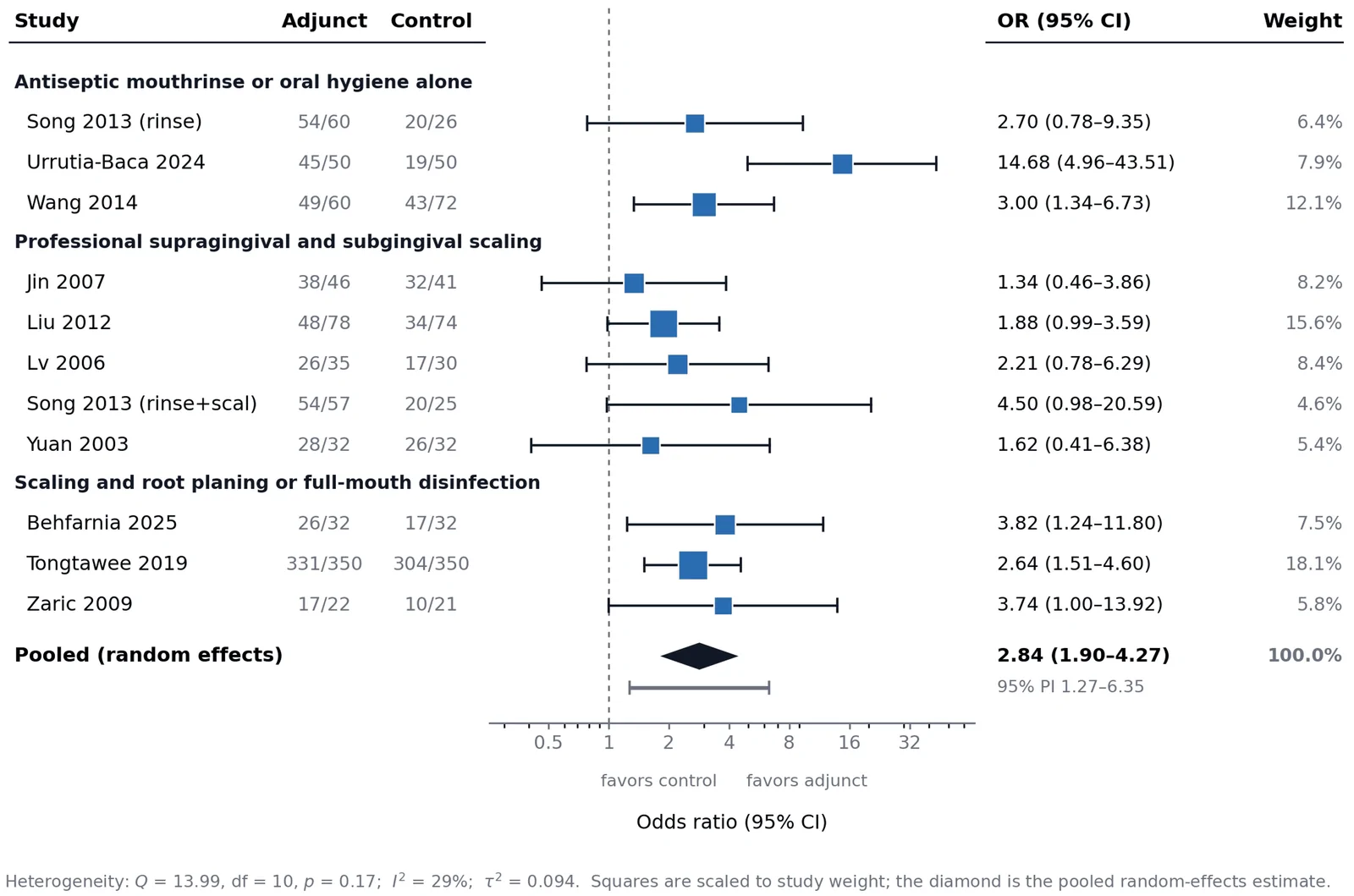
Forest plot for eradication stratified by the intensity of the adjunctive oral intervention. The comparisons shown, in the order plotted, are as follows. Antiseptic mouth rinse or oral hygiene alone: Song 2013, rinse arm [25]; Urrutia-Baca 2024 [20]; Wang 2014 [31]. Professional supragingival and subgingival scaling: Jin 2007 [28]; Liu 2012 [30]; Lv 2006 [27]; Song 2013, rinse plus scaling arm [25]; Yuan 2003 [26]. Scaling and root planing or full-mouth disinfection: Behfarnia 2025 [19]; Tongtawee 2019 [18]; Zaric 2009 [22]. The two intervention arms of Song 2013 [25] are entered separately, with the shared control group divided between them, so that 10 trials contribute 11 comparisons. Squares are scaled to study weight; the pooled diamond is shown for reference and is not the primary estimate, and the horizontal bar below it is the 95% prediction interval.

The single most informative comparison on this point comes from the one trial that used a matched inert placebo rinse [20]. Participants were allocated to an antiseptic mouth rinse or to a saline rinse within two strata, and every participant in the periodontal-disease stratum received scaling and root planing regardless of allocation, so the randomized contrast isolates the rinse. Eradication was achieved in 21 of 25 (84%) versus five of 25 (20%) among participants without periodontal disease and in 24 of 25 (96%) versus 14 of 25 (56%) among those with periodontal disease who had all received periodontal treatment, giving a pooled odds ratio of 14.68 (95% confidence interval 4.96 to 43.51). An antiseptic rinse with no mechanical component thus produced the largest effect in the dataset and produced it on top of periodontal therapy rather than instead of it.

Geographical origin did not explain the effect. The six trials conducted in China gave an odds ratio of 2.19 (95% confidence interval 1.35 to 3.54) with no heterogeneity, and the four conducted elsewhere gave 4.57 (95% confidence interval 1.29 to 16.23) with moderate heterogeneity. Trials published before the Cochrane search closed gave 2.27 (95% confidence interval 1.46 to 3.54), and those published afterwards gave 4.94 (95% confidence interval 0.52 to 46.86); the newer evidence therefore points in the same direction but is too few and too dispersed to give a precise estimate on its own. Restricting the analysis to the four trials whose arm-level counts were read from a directly obtained primary report gave 4.35 (95% confidence interval 1.31 to 14.39), larger than the overall estimate; the pooled result is therefore not an artifact of reliance on trials extracted from the Cochrane report. Excluding the two high-risk trials that contributed to this outcome gave 2.40 (95% confidence interval 1.68 to 3.43) with no heterogeneity, so the benefit persists, somewhat attenuated, among the better-conducted trials.

Small-Study Effects

The funnel plot for the eradication outcome was broadly symmetrical apart from one small trial with a very large effect (Figure 6). The Egger regression intercept was 0.95 (standard error 1.39; t = 0.68, df = 8; p = 0.51) and the Begg rank correlation gave a Kendall tau of 0.16 (p = 0.60). The trim-and-fill procedure, applied in the direction that publication bias would produce, in which small trials showing no benefit are presumed missing from the left of the funnel, imputed no studies and left the pooled estimate unchanged. These results are presented in Table 3. The positive Egger intercept is in the direction expected if small trials overstate benefit, but it is smaller than its standard error, and the Begg correlation was weak; consistent with this, trim-and-fill imputed no trials, so the adjusted estimate equals the primary one. We had anticipated, on the basis of the effect size and the composition of the literature, that small-study effects would be detectable; they were not. This should be interpreted with the caution that these tests have low power with 10 trials, but the available evidence does not support the specific concern that the pooled effect is an artifact of selective publication of small positive trials.

**Figure 6:**
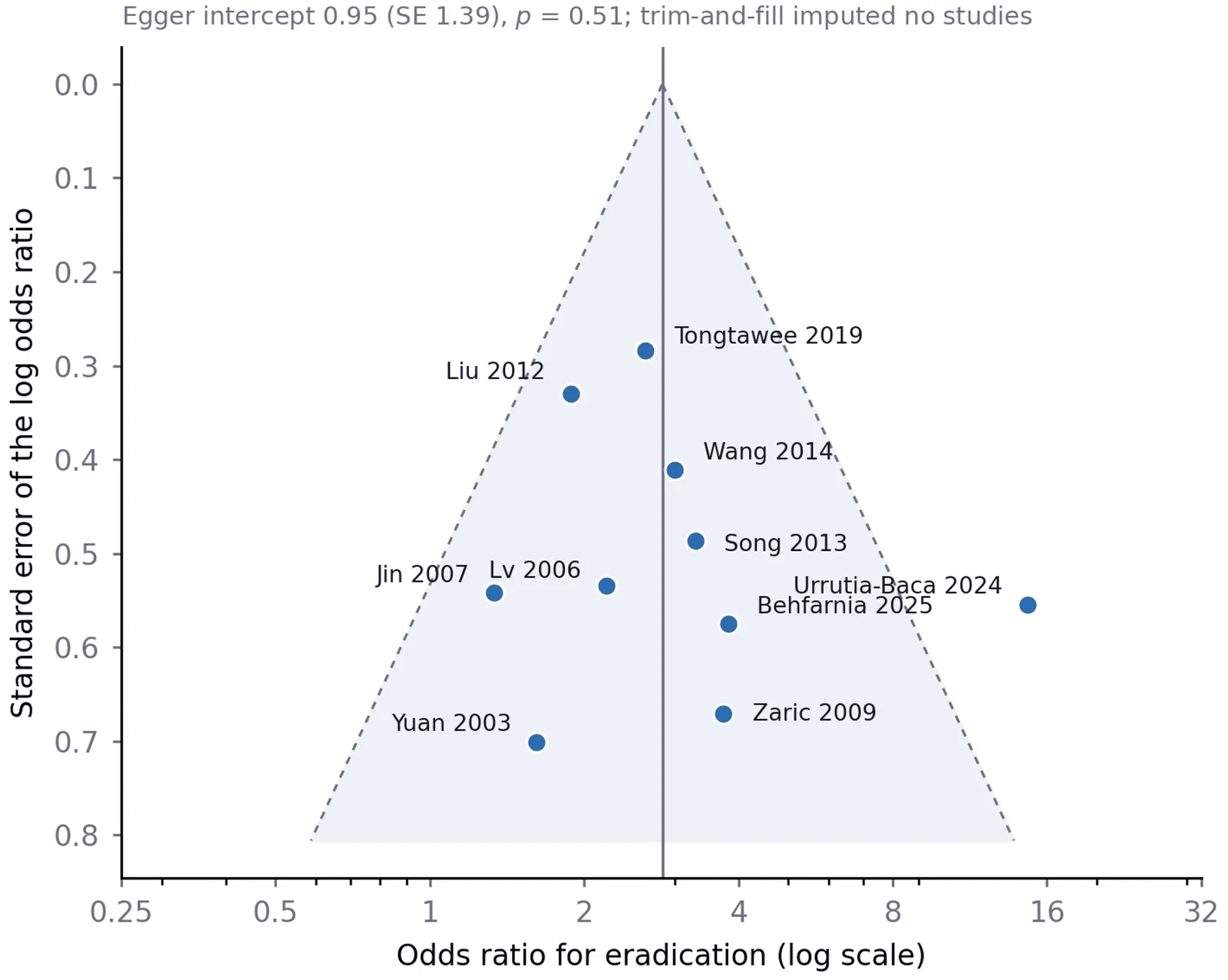
Funnel plot for the eradication outcome, with the 95% pseudo-confidence region. Ten of the 11 included trials are plotted rather than 11, because this funnel plot is drawn for the eradication outcome and Jia 2009 [29] contributed to the recurrence outcome only and reported no eradication data. The trials plotted are Yuan 2003 [26], Lv 2006 [27], Jin 2007 [28], Zaric 2009 [22], Liu 2012 [30], Song 2013 [25], Wang 2014 [31], Tongtawee 2019 [18], Urrutia-Baca 2024 [20] and Behfarnia 2025 [19]; the two intervention arms of Song 2013 [25] are combined into a single trial-level estimate, as in Figure 3. The Egger regression intercept was 0.95 (standard error 1.39, p = 0.51), the Begg rank correlation gave a Kendall tau of 0.16 (p = 0.60), and the trim-and-fill procedure imputed no missing studies; these tests are set out in Table 3.

Secondary Outcomes

Secondary outcomes were reported too sparsely to pool. Clearance of oral *H. pylori *was reported by three trials, each using a different and non-comparable method. Song 2013 [25] retested saliva with a rapid antigen assay four weeks after therapy and reported oral positivity of 76.5% with eradication therapy alone, 53.3% with the added mouth rinse, and 50.9% with mouth rinse and periodontal treatment. Wang 2014 [31] combined a salivary urease-antigen test, a salivary flagellin-antigen test, and polymerase chain reaction on both saliva and dental plaque, reporting clearance of 72.58% with the mouthwash against 10.67% with eradication therapy alone, but did not state when the mouth was re-sampled. Urrutia-Baca 2024 [20] re-tested subgingival dental plaque by quantitative polymerase chain reaction at four and 16 weeks and reported oral status as positive or negative for each of its four groups. Two further trials measured oral *H. pylori* at baseline without producing comparative clearance data: Behfarnia 2025 [19] re-tested subgingival plaque by rapid urease test one month after therapy in the periodontal-therapy arm only, so no between-arm comparison is possible, and for Zaric 2009 [22] the primary report could not be obtained, and a post-treatment oral result appears only in a secondary extraction that we could not verify. The remaining trials either did not sample the mouth or sampled it at baseline only. Periodontal clinical parameters, the natural outcome of a periodontal intervention, were defined in the methods of at least one trial [18] but were not reported in a poolable form by any trial. No trial reported a validated dyspepsia symptom score, and adverse events were not systematically reported in any trial.

Certainty ratings and the reasons for each judgment are summarized in Table 4. Certainty was moderate for eradication, downgraded once for risk of bias. Indirectness was considered because most trials used clarithromycin-based triple therapy but was not judged serious enough for a further downgrade: the adjunct was compared against the regimen that was standard in each trial's setting, and every trial favored the adjunct, including the two that used non-bismuth concomitant or bismuth quadruple regimens [19,20]. Certainty was low for absence of recurrence, downgraded once for risk of bias and once for imprecision: only four trials contributed, the prediction interval was very wide, and the risk-ratio confidence interval included no effect, so that statistical significance depended on the choice of effect measure. The substantial heterogeneity on the risk-ratio scale was considered under inconsistency but was not used for a separate downgrade, because it reflects the same scale dependence and would otherwise have been counted twice.

**Table 4:** Summary of findings and certainty of the evidence. Population: adults and children receiving eradication therapy for confirmed gastric *Helicobacter pylori *infection. Intervention: adjunctive periodontal or oral hygiene treatment. Comparison: eradication therapy alone. Certainty was rated with the Grading of Recommendations Assessment, Development and Evaluation (GRADE) approach [24]; certainty was moderate for eradication (downgraded for risk of bias) and low for absence of recurrence (downgraded for risk of bias and imprecision); outcomes that were not pooled were not rated. The assumed risk is the observed pooled control-group risk, and the corresponding risk with the adjunct is derived from that assumed risk together with the pooled odds ratio. The Reference column gives the reference numbers of the trials contributing to each outcome.

Outcome	Assumed risk with eradication therapy alone	Corresponding risk with the adjunct (95% confidence interval)	Relative effect (95% confidence interval)	Number of participants	Number of trials	Reference	Certainty of the evidence (GRADE)	Reasons for the certainty rating
Eradication, first post-treatment assessment (4 weeks to 3 months)	720 per 1,000	880 per 1,000 (826 to 919)	Odds ratio 2.84 (1.84 to 4.39)	1,575	10	[18-20,22,25-28,30,31]	Moderate	Downgraded once for risk of bias: no trials at low overall risk and three at high risk. Inconsistency not serious (I-squared 34%; prediction interval 1.18 to 6.87). Indirectness considered but not serious: most trials used clarithromycin-based triple therapy, but all 10 trials favored the adjunct, including those using concomitant or bismuth quadruple regimens [19,20]. Imprecision not serious. Small-study effects not detected, with low power (Table 3).
Absence of recurrence, 6 to 12 months	714 per 1,000	921 per 1,000 (837 to 963)	Odds ratio 4.64 (2.06 to 10.45); risk ratio 1.63 (0.86 to 3.09)	999	4	[18,26,28,29]	Low	Downgraded once for risk of bias, including one trial at high risk [29]. Downgraded once for imprecision: four trials, prediction interval 1.07 to 20.16, and the risk-ratio interval includes no effect, so statistical significance depends on the effect measure. Heterogeneity on the risk-ratio scale (I-squared 74%) is considered under inconsistency but not downgraded separately, as it reflects the same scale dependence. Indirectness as for eradication. Small-study effects not assessable with four trials.
Oral *Helicobacter pylori* clearance	Not estimable	Not estimable	Not estimable	Not estimable	3	[20,25,31]	Not rated	Not pooled: three different, non-comparable detection methods and sampling times.
Periodontal clinical parameters	Not estimable	Not estimable	Not estimable	Not estimable	0	Not applicable	Not rated	Not reported in a poolable form by any trial.
Dyspepsia symptoms	Not estimable	Not estimable	Not estimable	Not estimable	0	Not applicable	Not rated	No trial used a validated symptom score.
Adverse events	Not estimable	Not estimable	Not estimable	Not estimable	0	Not applicable	Not rated	Not systematically reported in any trial.

Discussion

Principal Findings

Across 11 randomized trials, adding an oral intervention to standard eradication therapy was associated with higher eradication and with a greater probability of remaining uninfected. The eradication estimate is stable in the ways that matter: the prediction interval excludes the null, the confidence interval excludes the null after the omission of any single trial, the estimate survives restriction to independently retrieved reports and to trials from outside China, and it survives exclusion of the trials at high risk of bias. Two prior syntheses reported comparable estimates from smaller evidence bases [15,16], and the trials published since the Cochrane search reproduce rather than attenuate the effect.

Two findings, however, complicate the straightforward reading. The first is that no dose-response gradient was detected. If adjunctive periodontal therapy works because it mechanically removes an oral reservoir of *H. pylori,* then full-mouth disinfection with subgingival instrumentation would be expected to outperform a twice-daily mouth rinse by a wide margin. The data do not show this. The three intensity strata overlap, the test for subgroup differences is not significant, and the ordering of the point estimates is, if anything, the reverse of the predicted one, with the least intensive category numerically the highest. The clearest single observation is the placebo-controlled rinse trial, in which an antiseptic mouth rinse with no mechanical component produced the largest effect in the whole dataset, and produced it in addition to scaling and root planing in the stratum that received it [20]. These comparisons rest on few trials with wide confidence intervals and cannot exclude a modest gradient; what they show is that the data provide no support for the gradient that the mechanical hypothesis predicts. The finding therefore bears on the proposed mechanism rather than on the intervention itself. The second is that the recurrence estimate, which is the outcome most often cited in support of the reservoir model, is fragile. It is significant on the odds ratio scale and not significant on the risk ratio scale, and heterogeneity rises from 20% to 74% between the two. An effect that changes its statistical status with the choice of effect measure should not be described as established.

Effect Without Evidence for the Proposed Mechanism

The reservoir hypothesis makes a specific molecular prediction: that the organisms recovered from the mouth and the stomach of the same patient are the same organisms. The most direct test of that prediction found otherwise. In a study of 21 patients with early gastric cancer, oral *H. pylori* DNA was detected in every patient at one or more oral sites, yet multilocus sequence typing matched the gastric sequence type in only one of the 21, with two further patients showing phylogenetic similarity [32]. If oral and gastric populations are usually genotypically distinct, the mouth is unlikely to be the usual source of the organism that recolonizes the stomach, and the rationale that has driven this line of trials requires re-examination, although a single study of 21 patients cannot settle the question.

Several alternative explanations are consistent with both the observed benefit and the lack of a detectable gradient. The first is chemical rather than mechanical. Chlorhexidine has direct activity against *H. pylori* in vitro: among five commercial oral rinses tested against clinical isolates, 0.2% chlorhexidine was the most effective, suppressing growth for 24 hours after only five to 30 seconds of exposure and downregulating cagA expression [33]. Chlorhexidine, or a comparable antiseptic, is featured in most of the included trials, including all three interventions in the least intensive stratum, and a proportion of any rinse is swallowed. The placebo-controlled trial of neutral electrolyzed water is the closest existing test of this pathway, since its comparator was an inert saline rinse delivered on the same schedule, and the agent it used has documented anti-biofilm activity against *H. pylori* in vitro [20]. That trial was small, open-label, and allocated by a predictable sequence, so it cannot carry the argument alone, but no larger trial has been designed with the chemical mechanism in mind, and the hypothesis remains untested at scale.

The second is behavioral. Adherence is the single strongest determinant of eradication success: in 38,698 patients in the European Registry on *H. pylori* Management, eradication was achieved in 89% of compliant patients compared with 58% of non-compliant patients, and adequate compliance was the variable most strongly associated with success in multivariable analysis, with an odds ratio of 5.9 (95% confidence interval 4.9 to 7.0) [34]. Adjunctive oral care entails additional clinical contacts, instruction, and monitoring, all of which plausibly improve adherence to a demanding multidrug regimen, and trials of a visible, effortful co-intervention cannot blind participants. An adherence effect of even a fraction of that magnitude would be sufficient to generate the pooled estimate we observed.

The third is confounding by phenotype. Untreated dental caries independently predicts eradication failure, with a dose-response across the number of decayed teeth, in a cohort in which periodontal treatment was not the exposure [35]. Oral disease may therefore mark a broader phenotype encompassing health behavior and adherence, rather than acting as a bacterial source.

The candidate pathways, the prediction that distinguishes each of them, and the evidence currently bearing on each are summarized in Figure 7. Distinguishing these explanations matters clinically. If the mechanism is chemical or behavioral rather than mechanical, the effective and scalable intervention may be an antiseptic mouth rinse and structured hygiene advice rather than a course of periodontal therapy, which is more costly, less available and, on the present evidence, not detectably more effective.

**Figure 7:**
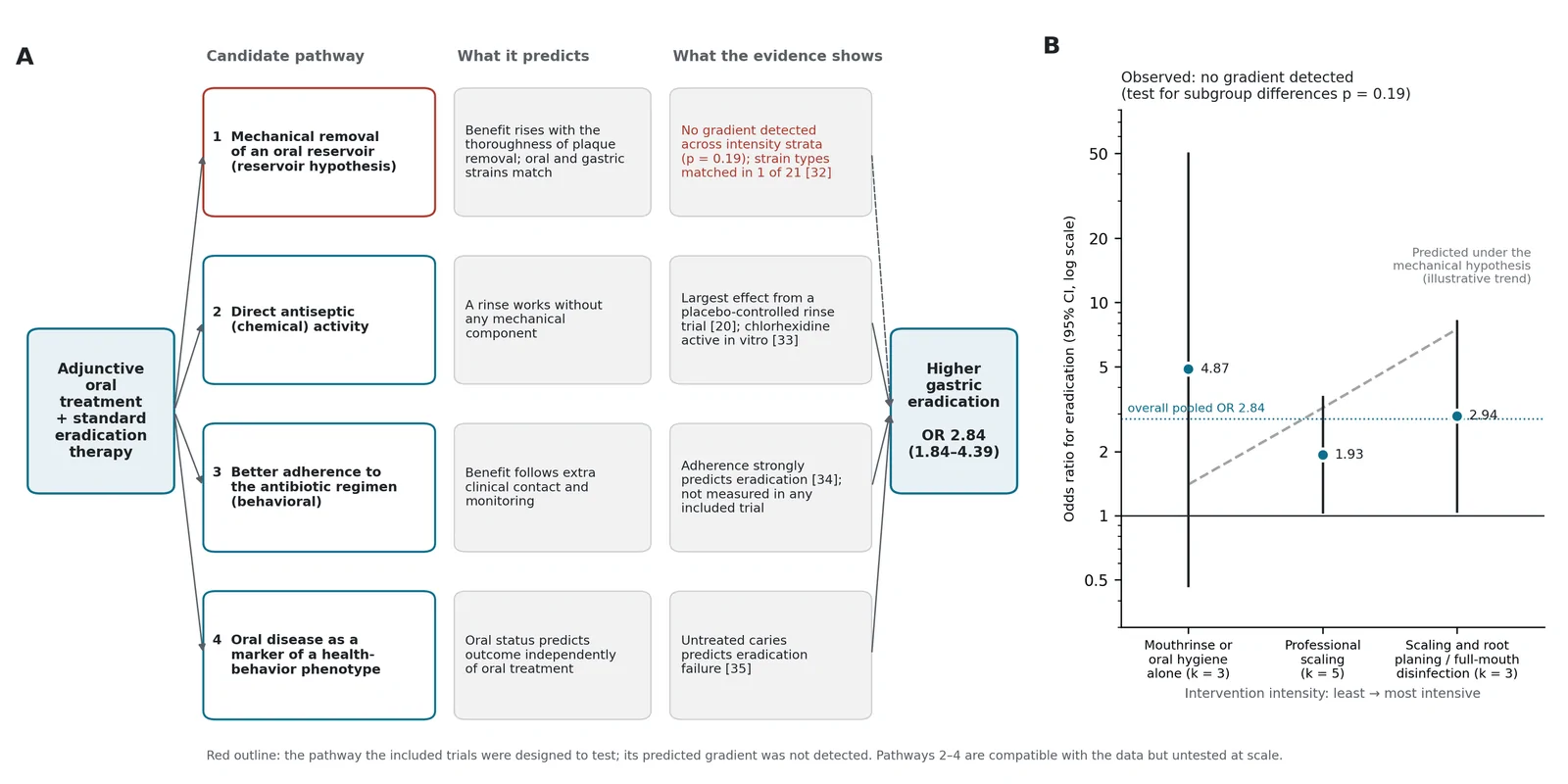
Candidate pathways linking adjunctive oral treatment to improved eradication of gastric Helicobacter pylori, and the intensity-stratified test of the mechanical reservoir hypothesis. (A) Four pathways compatible with the pooled benefit, with the prediction that distinguishes each and the evidence currently bearing on it: mechanical removal of an oral reservoir, the pathway the included trials were designed to test (red outline), with strain-typing evidence from [32]; direct antiseptic activity [20,33]; improved adherence to the antibiotic regimen [34]; and oral disease as a marker of a health-behavior phenotype [35]. (B) Pooled odds ratios with 95% confidence intervals for eradication in each intensity stratum, as in Figure 5 and Table 2; k, number of comparisons. The dashed line is an illustrative trend showing the pattern expected if benefit depended on the thoroughness of mechanical plaque removal and is not fitted to data; the dotted line is the overall pooled odds ratio of 2.84. The test for subgroup differences was not significant (p = 0.19), and the wide intervals mean that a modest gradient cannot be excluded.

Comparison With Previous Syntheses

Our eradication estimate of 2.84 is slightly larger than the Cochrane estimate of 2.15 from seven trials [15] and the 2021 update’s 2.65 from ten [16], and rests on a larger evidence base: 11 trials and 1,723 participants, against seven trials and 691 participants and 10 trials and 909 participants, respectively (Table 5). Restricted to the same trials those reviews had available, our estimate is 2.27; the increase comes from the three trials published since, and chiefly from one of them.

**Table 5:** Comparison with previous syntheses of the same question. Inchingolo 2025 [17] reported 11 included records rather than a defined number of randomized trials and did not report a pooled participant total, so no participant figure can be given for that review.

Synthesis	Search closed	Number of trials	Number of participants	Eradication	Recurrence	Language	Screening	Quantitative synthesis
Bouziane 2012 [14]	2011	3	298	Not pooled	Risk ratio of persistence 0.37 (0.21 to 0.64)	Not restricted	Duplicate	Yes
Ren 2016, Cochrane [15]	Aug.15	7	691	Odds ratio 2.15 (1.47 to 3.14)	Odds ratio 3.60 (2.11 to 6.15)	Not restricted	Duplicate	Yes
Ozturk 2021 [16]	2020	10	909	Odds ratio 2.65, p = 0.0002	Odds ratio 5.36, p = 0.0002	Not restricted	Not stated	Yes
Inchingolo 2025 [17]	Jan.25	11 records	Not stated	Not pooled	Not pooled	English only	Single reviewer	No
Present review	July 2026 (citation searching)	11	1,723	Odds ratio 2.84 (1.84 to 4.39)	Odds ratio 4.64 (2.06 to 10.45); risk ratio 1.63 (0.86 to 3.09)	Not restricted	Duplicate	Yes, with intensity stratification and small-study testing

The comparison with the most recent review is of a different kind. That review, registered as CRD42025644571, restricted eligibility to English-language reports published from 2015 onward, screened with a single reviewer, assessed randomized trials with an instrument designed for non-randomized studies, and planned a qualitative synthesis with no subgroup analyses [17]. Those choices exclude most of the randomized trials identified in the present review, and they explain why a 2025 review of this question could reach no quantitative conclusion. Our findings do not contradict it, but rather supply information it has not covered.

Limitations

Seven trials contributed arm-level data taken from the forest plots and characteristics tables of the Cochrane review rather than from their primary reports; six of these, including five Chinese-language reports, we were unable to obtain. These extractions are internally consistent and reproduce the published pooled totals exactly, and the sensitivity analysis restricted to trials whose counts came from a directly obtained primary report gave a larger rather than smaller effect; nonetheless, we could not independently verify the primary reports or reassess their risk of bias from source. For one trial, the denominators of the four arms were derived from reported percentages and numerators and were corroborated against the reported number of participants returning for evaluation [25].

One of the two non-Chinese trials taken from that source requires a specific declaration. The primary report of Wang 2014 [31] is available, but it reports percentages without denominators or event counts for any outcome. The counts recorded in the Cochrane forest plot for that trial, 49 of 60 and 43 of 72, do not appear in it and cannot be derived from it, whereas the percentages it does report imply 51 of 62 and 46 of 75, and its abstract and results section disagree with each other on the control-arm figure (61.33% against 61.54%). We have retained the Cochrane counts, both for consistency with the remainder of that extraction and because the review states that it extracted only the two arms meeting its inclusion criteria and corresponded with trial authors where necessary; the discrepancy may therefore reflect attrition data not published in the trial report. In a post hoc sensitivity analysis, substituting the counts implied by the primary report changes the pooled odds ratio from 2.84 (95% confidence interval 1.84 to 4.39) to 2.83 (95% confidence interval 1.83 to 4.38), with I-squared unchanged at 34%. The finding is therefore not sensitive to which set of counts is used, but readers should know that this trial’s contribution cannot be verified against its own published report.

The pooled eradication estimate is sensitive to one trial in a way the confidence interval does not fully convey. Omitting the placebo-controlled rinse trial reduces the estimate from 2.84 to 2.45 and removes all measurable heterogeneity [20]. That trial is the only one with a matched placebo comparator, which is a strength, but it is also small, open-label, allocated by a deterministic sequence, and reports an effect several times larger than any other trial in the field. Readers should treat the mouth rinse subgroup estimate in particular as hypothesis-generating.

A small number of reports identified only through the reference lists of previous syntheses could not be located or assessed. One citation we attempted to trace could not be found in any independent search, which is a caution about the accuracy of secondary reference lists in this field.

Eligible trials were identified by citation searching of three previous syntheses plus targeted searching for trials published since, rather than by an independent multi-database search. A trial missed by all three previous syntheses and not indexed in PubMed would therefore have been missed here, and the review inherits any selection idiosyncrasies of those syntheses. The review was not prospectively registered, so a reader cannot verify against a public record that the analyses reported here were those originally planned; the written protocol, the extracted dataset, and the full analysis code are provided in the supplementary material in mitigation. No trial was at low risk of bias; only one used a matched placebo comparator, none reported a validated symptom outcome, none reported adverse events systematically, and none reported periodontal outcomes in a poolable form, so we cannot say whether the periodontal intervention achieved its own local objectives. All trials come from five countries; none were conducted in Western Europe, the United States, Canada, or Africa, where baseline eradication rates, resistance patterns, and oral health differ. Most trials used clarithromycin-based triple therapy, which current guidance no longer recommends as empirical first-line treatment in high-resistance settings [5,6], so the applicability of these estimates to contemporary bismuth quadruple and susceptibility-guided regimens is uncertain. This indirectness was considered in the certainty assessment (Table 4); it did not lead to a further downgrade because every trial favored the adjunct, including the two that used non-bismuth concomitant or bismuth quadruple regimens [19,20], but it should be borne in mind when applying these estimates to current practice. Finally, tests for small-study effects have low power with 10 trials, and their negative result constrains but does not exclude concern.

Implications for Practice and Research

The evidence is sufficient to say that attention to the mouth during eradication therapy is associated with better outcomes, but not sufficient to say that periodontal therapy specifically is what produces the benefit. For patients with untreated periodontitis, there are independent reasons to treat it, and the present evidence adds a possible incidental gain. It does not justify recommending professional periodontal therapy solely as an adjunct to eradication, particularly where baseline eradication rates are high and the absolute benefit correspondingly small.

The trial that would settle this has not been done. It should be multicenter and conducted outside the regions that dominate the current evidence; it should use a contemporary bismuth quadruple or susceptibility-guided regimen; it should randomize participants to full periodontal therapy, to an antiseptic mouth rinse alone, and to a sham oral intervention matched for clinical contact time so that mechanical, chemical, and behavioral effects can be separated; it should measure adherence objectively rather than by self-report; it should follow participants for at least 12 months with a validated dyspepsia instrument and systematic adverse-event reporting; and it should genotype paired oral and gastric isolates so that the reservoir hypothesis is tested rather than presumed.

## Conclusions

Adding an oral intervention to standard eradication therapy for gastric *Helicobacter pylori* is associated with a higher eradication rate, with a pooled odds ratio of 2.84 and an absolute gain of about 16% at the observed control-group risk. The estimate remains significant after the exclusion of any single trial, after restriction to independently retrieved reports, and after exclusion of the trials at high risk of bias, and it is not attributable to small-study effects on the tests available. A parallel benefit for freedom from recurrence is present on the odds ratio scale but does not persist when the same trials are pooled as risk ratios and should not be treated as established.

The mechanism, however, may not be what the field has assumed. No increase in benefit with the thoroughness of mechanical plaque removal was detected, and an antiseptic mouthrinse alone performed at least as well as full-mouth disinfection, including in the one trial that compared an antiseptic rinse with a matched placebo rinse on top of periodontal therapy; although these subgroup comparisons had limited power, this sits alongside molecular evidence that oral and gastric strains within the same patient are usually genotypically distinct. Taken together, these observations argue that the benefit is unlikely to arise from mechanical elimination of an oral reservoir and more plausibly reflects direct antiseptic activity, improved adherence to a demanding regimen, or oral disease acting as a marker of a broader health-behavior phenotype. Until a trial separates those explanations, the reasonable clinical position is that oral hygiene deserves attention during eradication therapy, that periodontitis should be treated on its own merits, and that professional periodontal therapy should not yet be recommended specifically as an adjunct to *Helicobacter pylori *eradication.

## Figures and Tables

**Figure 8:**
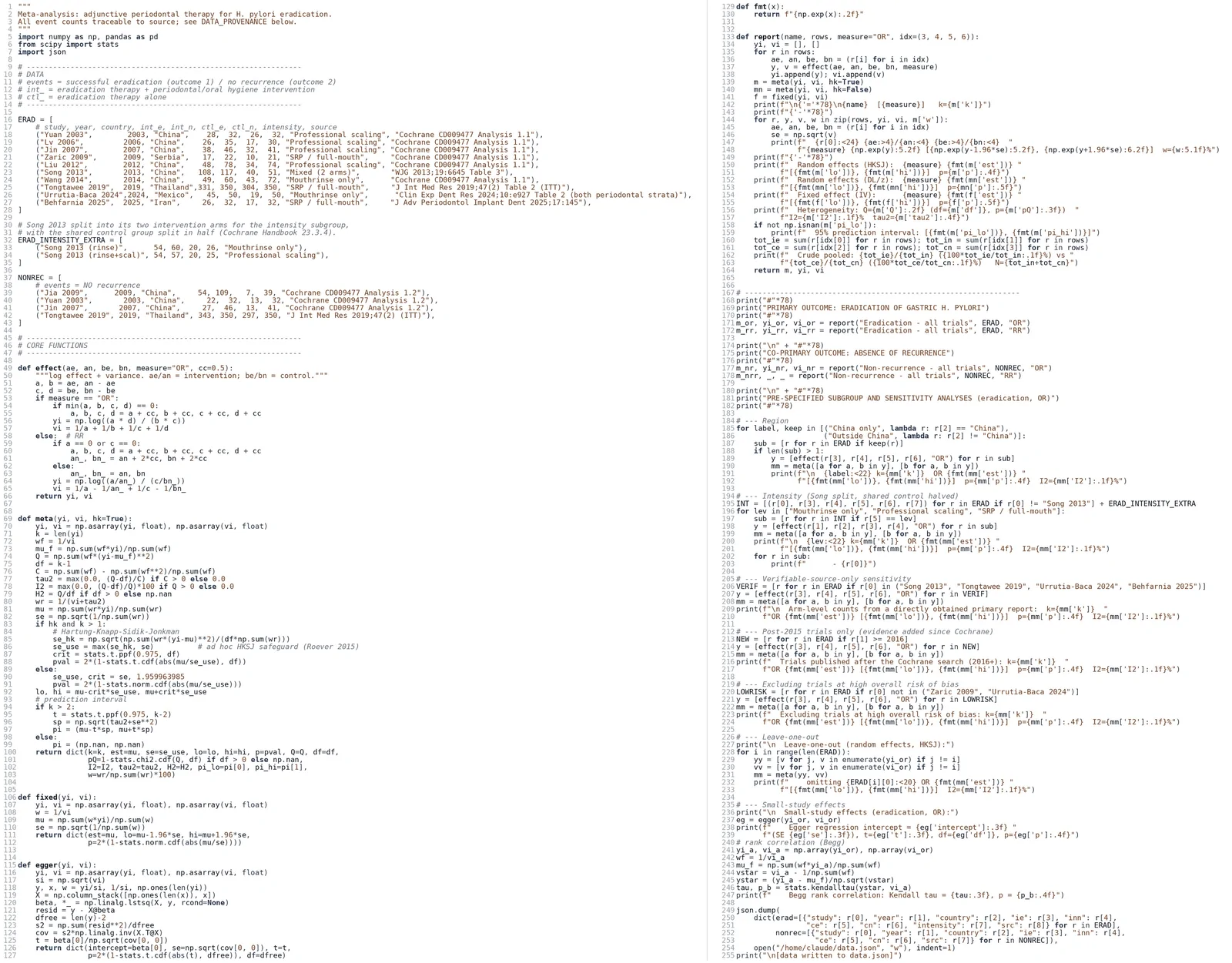
meta.py (data, effect measures, pooling, subgroups).

**Figure 9:**
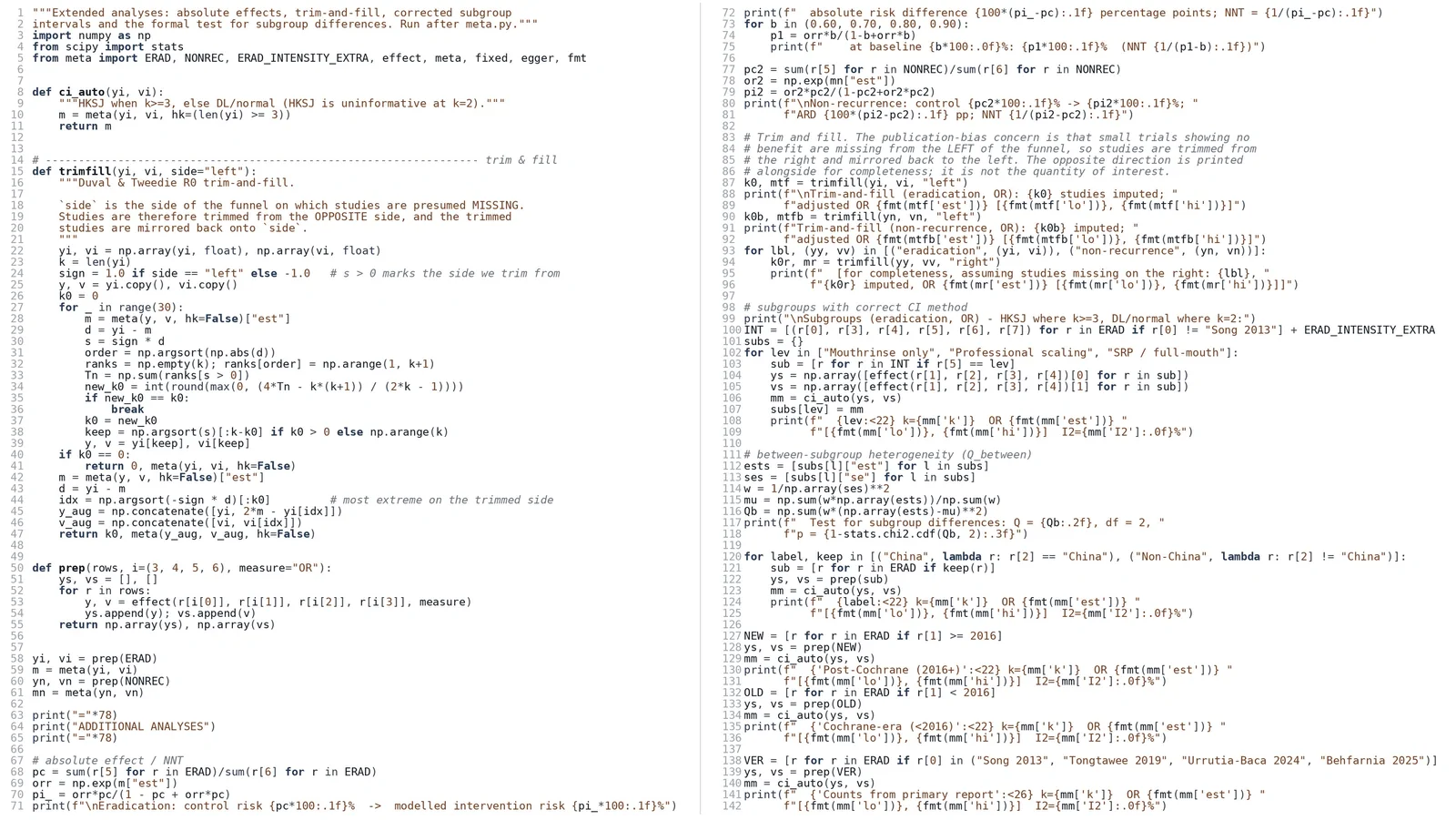
meta2.py (absolute effects, trim-and-fill, subgroup tests).

## Appendices

Appendix 1

PRISMA 2020 checklist

**Table 6:** PRISMA 2020 checklist.

Item	Topic	Checklist item	Location
	TITLE		
1	Title	Identify the report as a systematic review.	Title page
	ABSTRACT		
2	Abstract	See the PRISMA 2020 for Abstracts checklist.	Abstract
	INTRODUCTION		
3	Rationale	Describe the rationale in the context of existing knowledge.	Introduction, paragraphs 1-5
4	Objectives	Provide an explicit statement of the objective(s) or question(s).	Introduction, final paragraph
	METHODS		
5	Eligibility criteria	Specify inclusion and exclusion criteria and how studies were grouped.	Methods, Eligibility criteria
6	Information sources	Specify all sources searched and the date each was last searched.	Methods, Information sources and search
7	Search strategy	Present the full search strategy for all databases and registers.	Methods, Information sources and search — identification was by citation searching; no de novo database search was run
8	Selection process	Specify the methods used to decide whether a study met the criteria.	Methods, Study selection and data extraction
9	Data collection process	Specify the methods used to collect data from reports.	Methods, Study selection and data extraction
10a	Data items (outcomes)	List and define all outcomes for which data were sought.	Methods, Outcomes
10b	Data items (other)	List and define all other variables for which data were sought.	Methods, Study selection and data extraction; Table 1
11	Study risk of bias assessment	Specify the methods used to assess risk of bias.	Methods, Risk of bias and certainty of evidence; S4
12	Effect measures	Specify for each outcome the effect measure used.	Methods, Statistical analysis
13a	Synthesis methods	Describe processes used to decide which studies were eligible for each synthesis.	Methods, Statistical analysis
13b	Synthesis methods	Describe methods required to prepare the data for presentation or synthesis.	Methods, Statistical analysis; S2
13c	Synthesis methods	Describe methods used to tabulate or visually display results.	Methods, Statistical analysis; Figures 3-6
13d	Synthesis methods	Describe methods used to synthesise results and rationale for the choices.	Methods, Statistical analysis
13e	Synthesis methods	Describe methods to explore possible causes of heterogeneity.	Methods, Statistical analysis; Table 2
13f	Synthesis methods	Describe any sensitivity analyses conducted.	Methods, Statistical analysis; Table 2
14	Reporting bias assessment	Describe methods used to assess risk of bias due to missing results.	Methods, Statistical analysis; Results, Small-study effects; Table 3
15	Certainty assessment	Describe methods used to assess certainty in the body of evidence.	Methods, Risk of bias and certainty of evidence; Table 4
	RESULTS		
16a	Study selection	Describe the results of the search and selection process.	Results, Study selection; Figure 1
16b	Study selection	Cite studies that met the criteria but were excluded, and explain why.	S5
17	Study characteristics	Cite each included study and present its characteristics.	Table 1; S2
18	Risk of bias in studies	Present assessments of risk of bias for each included study.	Figure 2; S4
19	Results of individual studies	Present, for all outcomes, summary statistics for each group and an effect estimate.	Figures 3-5; S2
20a	Results of syntheses	Summarise the characteristics and risk of bias among contributing studies.	Results, Risk of bias
20b	Results of syntheses	Present results of all statistical syntheses.	Results; Figures 3-5
20c	Results of syntheses	Present results of all investigations of possible causes of heterogeneity.	Results, Subgroup and sensitivity analyses; Table 2
20d	Results of syntheses	Present results of all sensitivity analyses.	Table 2
21	Reporting biases	Present assessments of risk of bias due to missing results.	Results, Small-study effects; Figure 6; Table 3
22	Certainty of evidence	Present assessments of certainty for each outcome assessed.	Table 4
	DISCUSSION		
23a	Discussion	Provide a general interpretation of the results in the context of other evidence.	Discussion, Principal findings; Comparison with previous syntheses
23b	Discussion	Discuss any limitations of the evidence included.	Discussion, Limitations
23c	Discussion	Discuss any limitations of the review processes used.	Discussion, Limitations
23d	Discussion	Discuss implications for practice, policy and future research.	Discussion, Implications for practice and research
	OTHER INFORMATION		
24a	Registration and protocol	Provide registration information, including the register name and number.	Methods, Protocol, registration and reporting — the review was not registered in a prospective register
24b	Registration and protocol	Indicate where the protocol can be accessed, or state it was not prepared.	A written protocol was prepared before data were pooled but not publicly posted; the analysis plan it contains is reproduced in full in the Methods and in S2 and S6
24c	Registration and protocol	Describe and explain any amendments to the registered protocol.	None; the eligibility criteria, outcomes and analysis plan were not altered after data were pooled
25	Support	Describe sources of financial or non-financial support.	Additional information, Disclosures
26	Competing interests	Declare any competing interests of review authors.	Additional information, Disclosures
27	Availability of data, code	Report which of the following are publicly available and where.	S2; S6

Appendix 2

Extracted Dataset and Provenance of Every Number

**Table 7:** Extracted dataset and provenance of every number. Events are the number of participants achieving the outcome; totals are the number analyzed in that arm. The final column records exactly where each pair of counts came from.

Outcome	Trial	Country	Adjunct events / n	Control events / n	Intensity stratum	Provenance
Eradication	Yuan 2003 [26]	China	28 / 32	26 / 32	Professional scaling	Read from the published forest plot of Analysis 1.1 of the Cochrane review. The six trials taken from this source sum to 206/273 and 162/270 and reproduce the published pooled odds ratio of 2.15 exactly (see S3).
Eradication	Lv 2006 [27]	China	26 / 35	17 / 30	Professional scaling	Read from the published forest plot of Analysis 1.1 of the Cochrane review. The six trials taken from this source sum to 206/273 and 162/270 and reproduce the published pooled odds ratio of 2.15 exactly (see S3).
Eradication	Jin 2007 [28]	China	38 / 46	32 / 41	Professional scaling	Read from the published forest plot of Analysis 1.1 of the Cochrane review. The six trials taken from this source sum to 206/273 and 162/270 and reproduce the published pooled odds ratio of 2.15 exactly (see S3).
Eradication	Zaric 2009 [22]	Serbia	17 / 22	10 / 21	Scaling and root planing / full-mouth	Read from the published forest plot of Analysis 1.1 of the Cochrane review. The six trials taken from this source sum to 206/273 and 162/270 and reproduce the published pooled odds ratio of 2.15 exactly (see S3).
Eradication	Liu 2012 [30]	China	48 / 78	34 / 74	Professional scaling	Read from the published forest plot of Analysis 1.1 of the Cochrane review. The six trials taken from this source sum to 206/273 and 162/270 and reproduce the published pooled odds ratio of 2.15 exactly (see S3).
Eradication	Song 2013 [25]	China	108 / 117	40 / 51	Mixed (2 arms)	Numerators and percentages read directly from the primary report; denominators back-calculated from the reported percentages (45, 51, 60, 57) and corroborated against the stated 213 participants returning for evaluation from 233 enrolled. The trial randomized only the 180 participants who were positive for oral *H. pylori,* into triple therapy alone (51 evaluated, 78.4%), triple therapy plus mouthrinse (60 evaluated, 90.0%) and triple therapy plus mouthrinse and periodontal treatment (57 evaluated, 94.7%); the two intervention arms are combined here as 108 of 117. The remaining group of 45 evaluated participants was defined by a negative oral *H. pylori *test rather than by randomization and is excluded.
Eradication	Wang 2014 [31]	China	49 / 60	43 / 72	Mouthrinse only	Read from the published forest plot of Analysis 1.1 of the Cochrane review. The six trials taken from this source sum to 206/273 and 162/270 and reproduce the published pooled odds ratio of 2.15 exactly (see S3). These counts cannot be reproduced from the primary report, which is available but gives percentages without denominators or event counts; its own percentages imply 51 of 62 and 46 of 75, and its abstract and results section disagree on the control-arm figure. Substituting the counts implied by the primary report changes the pooled odds ratio from 2.84 to 2.83. See Limitations.
Eradication	Tongtawee 2019 [18]	Thailand	331 / 350	304 / 350	Scaling and root planing / full-mouth	Read directly from Table 2 of the primary report, intention-to-treat rows. Note the report is internally inconsistent: the text states 347 and 342 randomized while the intention-to-treat denominators are 350 and 350.
Eradication	Urrutia-Baca 2024 [20]	Mexico	45 / 50	19 / 50	Mouthrinse only	Read directly from Table 2 of the primary report. The trial randomized participants to an active mouthrinse or a saline placebo rinse within two strata defined by the presence or absence of periodontal disease; every participant in the periodontal-disease stratum received scaling and root planing regardless of allocation. Only the randomized rinse contrast is used, pooled across strata: 21/25 versus 5/25 without periodontal disease and 24/25 versus 14/25 with it. The trial’s between-strata comparison of periodontal treatment is not randomized and is not entered into any analysis.
Eradication	Behfarnia 2025 [19]	Iran	26 / 32	17 / 32	Scaling and root planing / full-mouth	Derived by subtraction from the verbatim statement that positive stool antigen results were observed in 6 of group 1 and 15 of group 2, each of 32 randomized participants. The trial also reports a third group of 38 participants who tested negative for oral *H. pylori* and received bismuth quadruple therapy alone; that group was defined by infection status rather than by randomization and is excluded. Note the printed percentages (80% and 52%) differ slightly from the derived counts (81.3% and 53.1%).
Non-recurrence	Jia 2009 [29]	China	54 / 109	7 / 39	not applicable	Read from the published forest plot of Analysis 1.2 of the Cochrane review. The three trials taken from this source sum to 103/187 and 33/112 and reproduce the published pooled odds ratio of 3.60 exactly (see S3).
Non-recurrence	Yuan 2003 [26]	China	22 / 32	13 / 32	not applicable	Read from the published forest plot of Analysis 1.2 of the Cochrane review. The three trials taken from this source sum to 103/187 and 33/112 and reproduce the published pooled odds ratio of 3.60 exactly (see S3).
Non-recurrence	Jin 2007 [28]	China	27 / 46	13 / 41	not applicable	Read from the published forest plot of Analysis 1.2 of the Cochrane review. The three trials taken from this source sum to 103/187 and 33/112 and reproduce the published pooled odds ratio of 3.60 exactly (see S3).
Non-recurrence	Tongtawee 2019 [18]	Thailand	343 / 350	297 / 350	not applicable	Recurrence figures read directly from the primary report (2.0% and 15.14%), with the whole arm as the denominator, matching the denominator convention used by the Cochrane review.

The Song 2013 trial contributes two intervention arms sharing one control group. For the intensity-stratified analysis, the arms are entered separately as 54 of 60 (mouthrinse alone) and 54 of 57 (mouthrinse with scaling), with the shared control group of 40 of 51 divided as 20 of 26 and 20 of 25, respectively, following Cochrane Handbook section 23.3.4.

The Urrutia-Baca 2024 trial has four groups but only one randomized contrast, the active mouthrinse against the saline placebo rinse, replicated within two periodontal strata. It therefore enters as a single two-arm comparison of 45 of 50 against 19 of 50 and is allocated wholly to the mouthrinse-only intensity stratum. Its recurrence data were collected 16 weeks after the end of therapy, short of the prespecified minimum of six months, so the trial contributes to the eradication outcome only.

Appendix 3

Extraction-Fidelity Check Against the Published Cochrane Results

**Table 8:** Extraction-fidelity check against the published Cochrane results. Because most arm-level counts were transcribed from a published review rather than read from primary reports, the transcription was checked by reproducing that review’s own published results before any new analysis was run. The six trials whose eradication data were transcribed from Analysis 1.1 of the Cochrane review were pooled in isolation using the same fixed-effect Mantel-Haenszel method that review used. The transcription reproduces the published values exactly. Each cell in the last two rows contains a single number, an odds ratio, written with a decimal point: the value published by Ren 2016 [15] is given to two decimal places as published, and the value recomputed from our extraction is given to four decimal places, so that the agreement between them can be judged exactly. In the previous version, these odds ratios were rendered with a decimal comma, which made each look like two numbers separated by a comma; this has been corrected throughout.

Quantity	Published by Ren 2016 [15]	Recomputed from our extraction
Eradication, adjunct arm events / total	206 / 273	206 / 273
Eradication, control arm events / total	162 / 270	162 / 270
Eradication, pooled odds ratio	2.15	2.1494
Non-recurrence, adjunct arm events / total	103 / 187	103 / 187
Non-recurrence, control arm events / total	33 / 112	33 / 112
Non-recurrence, pooled odds ratio	3.6	3.5998

Appendix 4

Risk of Bias, Domain-Level Judgments

**Table 9:** Risk of bias, domain-level judgments. Assessed with the second version of the Cochrane risk-of-bias tool [23]. D1: randomization process; D2: deviations from intended interventions; D3: missing outcome data; D4: measurement of the outcome; D5: selection of the reported result. Supporting statements for the five trials whose primary reports are in Chinese are taken from the characteristics-of-included-studies table of the Cochrane review [15]; the remainder are from the primary reports.

Trial	D1	D2	D3	D4	D5	Overall	Supporting statement
Yuan 2003 [26]	Some concerns	Some concerns	Low	Low	Some concerns	Some concerns	Sequence generation and allocation concealment not described. No blinding described. All participants followed to the end of the trial with an intention-to-treat analysis. Outcome is a carbon-14 urea breath test, an objective laboratory measure. No prespecified protocol available.
Lv 2006 [27]	Some concerns	Some concerns	Low	Low	Some concerns	Some concerns	Sequence generation and allocation concealment not described. No blinding described. All participants followed; intention-to-treat analysis. Outcome by rapid urease test or histology. No protocol available.
Jin 2007 [28]	Some concerns	Some concerns	Low	Low	Some concerns	Some concerns	Sequence generation and allocation concealment not described. No blinding described. All participants followed; intention-to-treat analysis. Carbon-14 urea breath test. No protocol available.
Zaric 2009 [22]	Some concerns	Some concerns	High	Low	Some concerns	High	Described as block randomized; the Cochrane review records that this was confirmed by correspondence with the trial authors. Allocation concealment not described. Outcome assessor blinded (single blind). An unstated number of participants from both groups did not complete the study, and neither the number nor the reasons were reported: this is a high risk of bias from missing outcome data and determines the overall judgment. Outcome by nested polymerase chain reaction.
Jia 2009 [29]	Some concerns	Some concerns	Low	Some concerns	Some concerns	High	Sequence generation and allocation concealment not described. All patient data reported, including non-adherent participants, with an intention-to-treat approach. However, every participant was reported as free of gastric infection after eradication therapy, an implausible 100% success rate that raises serious concern about outcome measurement and determines the overall judgment.
Liu 2012 [30]	Some concerns	Some concerns	Some concerns	Low	Some concerns	Some concerns	Sequence generation and allocation concealment not described. No blinding described. Sixteen participants lost to follow-up, though an intention-to-treat approach was used. Rapid urease test plus carbon-14 urea breath test.
Song 2013 [25]	Some concerns	Some concerns	Some concerns	Low	Some concerns	Some concerns	Described as randomized, but the method is not stated. Of 233 enrolled participants, the 180 positive for oral *H. pylori *were randomly allocated to triple therapy alone, triple therapy plus mouthrinse, or triple therapy plus mouthrinse and periodontal treatment; a fourth group of 53 enrolled (45 evaluated) who were negative for oral *H. pylori *was defined by infection status rather than by randomization and is excluded here. Twenty of the 233 enrolled did not return for evaluation (8.6%), and the analysis is effectively per-protocol. No blinding described. Carbon-13 urea breath test. Reported percentages without denominators, which required back-calculation.
Wang 2014 [31]	Some concerns	Low	Low	Low	Some concerns	Some concerns	Sequence generation and allocation concealment not described. Double blind, covering participants and outcome assessors, so deviations from intended interventions are at low risk. All participants followed to the end. Carbon-13 urea breath test.
Tongtawee 2019 [18]	Some concerns	Some concerns	Low	Low	Some concerns	Some concerns	Described as randomized, but the method of sequence generation is not stated. Participants could not be blinded to an oral intervention. Both intention-to-treat and per-protocol analyses reported with no unexplained attrition. Outcome confirmed by stool antigen, rapid urease test, histology, and polymerase chain reaction. The report contains an internal inconsistency between the randomized numbers (347 and 342) and the intention-to-treat denominators (350 and 350), which is a concern for the selection of the reported result.
Urrutia-Baca 2024 [20]	High	Some concerns	Low	Low	Some concerns	High	Allocation followed the order in which participants consented, with odd numbers assigned to the saline control rinse and even numbers to the active rinse. This sequence is deterministic and cannot be concealed, which is a high risk of bias in the randomization domain and determines the overall judgment. Open-label, with only the statisticians blinded. All 100 randomized participants are accounted for at the 4-week eradication endpoint. Outcome by quantitative polymerase chain reaction on stool, an objective laboratory measure. Ethics approval is reported, but no prespecified protocol or registration is cited.
Behfarnia 2025 [19]	Low	Low	Low	Low	Some concerns	Some concerns	Randomization and allocation described; double-masked, with a blinded outcome assessor. Of 102 participants analyzed, 64 who were positive for oral *H. pylori* were randomly allocated to periodontal therapy plus bismuth quadruple therapy (n = 32) or bismuth quadruple therapy alone (n = 32); a third group of 38 who were negative for oral *H. pylori* received bismuth quadruple therapy alone and was allocated by infection status rather than at random, so it is excluded here. Stool antigen enzyme immunoassay. The printed percentages (80% and 52%) differ slightly from the counts derived from the stated numbers of positive results (26 of 32 and 17 of 32), a minor concern for the selection of the reported result.

Summary: no trial at low overall risk of bias; eight with some concerns; three at high risk. The dominant recurring problems are undescribed sequence generation and allocation concealment, and the absence of a prespecified protocol against which the reported analysis could be checked.

Appendix 5

Studies Considered but Not Included, With Reasons

**Table 10:** Studies considered but not included, with reasons.

Study	Reason
Chen 2022, Chin J Microecol	Reported in secondary sources as a randomized trial comparing scaling and root planing (87.4%) with supragingival scaling (75.4%). Full text not retrievable; arm-level counts unobtainable.
Figliuzzi 2020	Cited only in the reference list of Inchingolo 2025 as a prospective study of 44 patients. Design (prospective, not randomized) and arm-level data could not be confirmed from an independent source.
Yadollahzadeh 2022	Cited only in the reference list of Inchingolo 2025. Could not be located in any independent search of PubMed, Crossref, Scopus or general web search. Not included.
Venkata 2021	Cited in Inchingolo 2025 as a prospective observational study. Excluded as non-randomized; would be eligible only for a separate secondary synthesis.
Jia 2012	Cited in Inchingolo 2025 as a study of professional plaque control in 56 versus 51 patients. Could not be distinguished with confidence from Jia 2009 and arm-level data were unobtainable.
Jia 2009b, J Periodontol 80:1606-9	Duplicate report of the same cohort as Jia 2009 (Hua Xi Kou Qiang Yi Xue Za Zhi), and excluded on that basis by the Cochrane review. Only the Chinese-language report is included here.
Xu 2016 / Xu 2018 (preschool children)	Trial of oral *H. pylori *clearance with no systemic eradication regimen and no gastric outcome; outside the review question. The two reports may describe the same cohort.
Xu 2001, Tarullo 2001, Hou 2002, Hou 2003, Hou 2004, Ierardi 1998, Yang 2013, Ye 2012, Qian 2014, Avcu 2001	Excluded by the Cochrane review for the reasons recorded in its characteristics-of-excluded-studies table: not randomized, no periodontal therapy administered, no gastric eradication outcome, or insufficient detail to extract. Reasons adopted here.

Appendix 6

Analysis Code

Python 3.11 with NumPy 2.4 and SciPy 1.17. The code is self-contained: the trial data are declared at the top of the first file, so running the two files in order reproduces every estimate reported in the manuscript from scratch. The plotting scripts that draw the figures from these same objects are available from the corresponding author on request.

Appendix 7
